# Supplementary material for: Evolving infectious disease dynamics shape school-based intervention effectiveness
Source: Nat Commun. 2025 Jul 17;16:6597. doi: 10.1038/s41467-025-61925-5 (PMC12271494; doi:10.1038/s41467-025-61925-5)
Supplement: Supplementary file 1 — Supplementary Information [file 41467_2025_61925_MOESM1_ESM.pdf]

Supplementary Material - Evolving infectious disease dynamics shape school-based intervention effectiveness

Javier Perez-Saez, Mathilde Bellon, Justin Lessler, Julie Berthelot, Emma Hodcroft, Grégoire Michielin, Francesco Pennacchio, Julien Lamour, Florian Laubscher, Arnaud L'Huillier, Klara M Posfay-Barbe, Sebastian J Maerckl, Idris Guessous, Andrew S Azman, Isabella Eckerle, Silvia Stringhini, Elsa Lorthe; for the SEROCoV-Schools study group

Contents

S1 Materials and methods **S3**
S1.1 Sequencing and phylogenetic analysis . . . S3
S1.2 Modeling analyses . . . S4
S1.2.1 Statistical modeling framework . . . S4
S1.2.2 Dynamic modeling framework . . . S7
S1.3 Scenario simulations . . . S13
S1.3.1 Main analysis . . . S13
S1.3.2 Sensitivity analyses . . . S13
S1.4 Data on school closure timings during the COVID-19 pandemic . . . S14

S2 Supplementary Figures **S15**
S2.1 Figure S1: Study flow chart . . . S15
S2.2 Figure S2: Stringency of non-pharmaceutical interventions in Switzerland . . . S16
S2.3 Figure S3: Longitudinal participant data . . . S17
S2.4 Figure S4: Sequence alignments . . . S18
S2.5 Figure S5: Statistical model fidelity to data . . . S19
S2.6 Figure S6: Inference of time-varying serology sensitivity . . . S20
S2.7 Figure S7: Statistical modeling inference of outbreak attack rates . . . S20
S2.8 Figure S8: School SARS-CoV-2 transmission model diagram . . . S21
S2.9 Figure S9: Natural history parameters . . . S21
S2.10Figure S10: Dynamic model fidelity to data . . . S22
S2.11Figure S11: Comparison of model predictions . . . S23
S2.12Figure S12: Scenario simulations sensitivity all results . . . S23
S2.13Figure S13: Scenario simulations sensitivity by feedback . . . S24
S2.14Figure S14: Scenario simulations sensitivity by duration . . . S24
S2.15Figure S15: Empirical data on transmission intensity during school closures . . . S25
S2.16Figure S16: Human mobility data for the state of Geneva . . . S26

S3 Supplementary Tables **S27**
S3.1 Table S1: Baseline children . . . S27
S3.2 Table S2: Baseline adults . . . S27
S3.3 Table S3: Alpha outbreaks . . . S28
S3.4 Table S4: Delta outbreaks . . . S29
S3.5 Table S5: Omicron BA.1 outbreaks . . . S30
S3.6 Table S6: GISAID sequences - Alpha . . . S31
S3.7 Table S7: GISAID sequences - Delta/Omicron BA.1 . . . S32
S3.8 Table S8: Parameter values of dynamic model . . . S33

S3.9 Table S9: Genbank accession numbers of sequences generated in this study S34
S3.10 Table S10: GISAID DOI of sequences generated in this study S35

S4 The SEROCoV-Schools study group **S36**

S1 Materials and methods

S1.1 Sequencing and phylogenetic analysis

SARS-CoV-2 whole genome sequencing was performed for positive samples at the Health 2030 Genome Center (Geneva) using the Illumina COVIDSeq library preparation reagents following the protocol provided by the supplier. Only the sequences with coverage higher than 90% were included in the present analysis (at least 27,000 bases). The consensus sequences were verified one by one by a bio-informatician and then submitted on GISAID (sequence list in Supplementary Figure S2).

All globally available SARS-CoV-2 sequences on GISAID were downloaded on 26 Apr 2023 [1]. The full dataset was reduced for computational efficiency to include only sequences up to 30 June 2022, covering the period through which samples were taken in the schools. This resulted in 12,076,698 sequences. 12 Alpha, 30 Delta (Nextstrain clade 21J; Pango lineage AY), and 36 Omicron (Nextstrain clade 21K; Pango lineage BA.1) near-full-length and good-coverage sequences from SEROCoV-Schools were included in the phylogenetic analysis. These sequences served as the 'focal' set of the analysis.

Using the cluster-focused workflow developed for CoVariants.org (a variant of the Nextstrain 'ncov' pipeline), phylogenies were generated, which included genetically closely related sequences to our 'focal set' [2]. The specific scripts, workflow, and configuration files used to generate these phylogenies can be found at https://github.com/emmahodcroft/ncov_2021/tree/geneva_school_analysis. Briefly, this aims to include a large number of sequences that are closely related to the focal set in order to contextualize the sequences with potential links outside of the study area. Two phylogenies were run, one focusing on Alpha, and the other on Delta and Omicron, separately purely due to the difference in time when the sequences became available. The phylogenies included all of the focal sequences, then aimed to add up to 6,000 genetically similar sequences to the focal set from around the globe, and 200 sequences spread evenly through time and geography to provide a 'backbone' to the phylogeny and enable accurate rate estimations, topology reconstruction, and appropriate rooting. For the Alpha phylogeny, the analysis was limited to only include sequences up to 10 June 2021, considerably after the last collected sample from SEROCoV-Schools on 1 May 2021. For the Delta and Omicron phylogeny, the analysis was limited to only include sequences up to 26 Feb 2022, considerably after the last collected Omicron sample of SEROCoV-Schools on 21 Jan 2022.

For Alpha, the final phylogenies included 10 of the 12 samples from our study (Switzerland/GE-CEVD-72015900/2021 and Switzerland/GE-CEVD-72016600/2021 were excluded due to not having at least 27,000 bases), an additional 1,963 genetically similar sequences from the canton of Geneva, and a further 3,183 genetically similar and background sequences from around the world, leading to phylogeny of 5,156 total sequences. The final set of sequences in the analysis can be found at 10.55876/gis8.240117ea.

For Delta and Omicron, the final phylogenies included 54 of the 66 samples from Geneva schools (10 sequences (Switzerland/GE-CEVD-72015200/2021, Switzerland/GE-CEVD-72028701/2021, Switzerland/GE-CEVD-72028710/2021, Switzerland/GE-CEVD-72001200/2021, Switzerland/GE-CEVD-72059500d/2021, Switzerland/GE-CEVD-72061900/2022, Switzerland/GE-CEVD-72064101/2022, Switzerland/GE-CEVD-72065700/2022, Switzerland/GE-CEVD-72066620/2022, Switzerland/GE-CEVD-65092502/2022) were excluded for being below 27,000 bases; Switzerland/GE-CEVD-72063500/2022 and Switzerland/GE-CEVD-72061501/2022 were excluded for being too poor quality), an additional 490 genetically similar sequences from the canton of Geneva, and a further 5,073 genetically similar and background sequences from around the world, leading to a phylogeny containing 5,617 total sequences. The final set of sequences in the analysis can be found at https://doi.org/10.55876/gis8.240117cm.

The alignments were performed with the software Geneious Prime with the focal set sequences. As a reference sequence, we used the first sequence of each variant (Alpha, Delta, and Omicron BA.1) obtained in Geneva, i.e., Switzerland/GE-32807087/2020, Switzerland/GE-HUG-34175853/2021 and Switzerland/GE-HUG-36200302/2021 (7). Of note, the sequences Switzerland/GE-CEVD-72016611/2021, Switzerland/GE-HUG-36485138/2021, Switzerland/GE-HUG-36122476/2021, and Switzerland/GE-HUG-36615497/2022 are not considered part of an outbreak investigation because they do not meet the criteria presented in Supplementary material and methods, section 3b. That is why they are not present in the alignment.

Sequence alignments are given in figure S4.

S1.2 Modeling analyses

The overall aim of the analysis is to determine the relative contributions of within-school transmission vs. community importation in the study population. We approach the problem through two modeling frameworks, first a statistical model and then a dynamic transmission model. We resort to two modeling frameworks to strengthen the robustness of the results, as well as to feed results from the statistical model into the the dynamic model. In particular we infer the time-varying sensitivity of the capillary blood serology used in this study in the statistical framework into the dynamic model framework. The robustness of the results was assessed through the triangulation of the predicted seroprevalence from both modeling frameworks together with our population-level estimates of seroprevalence in the state of Geneva at three time points during the pandemic [3, 4, 5].

Code for the statistical and dynamic modeling analyses are available at https://github.com/UEP-HUG/serocov-schools-public.

S1.2.1 Statistical modeling framework

The aim of the statistical modeling framework was to infer the time-varying sensitivity of capillary blood serology in our study population as well as to infer time changes in school-group level seroprevalence. The model tracks the individual-level probability of SARS-CoV-2 infection in time intervals characterized by constant infection hazard. We account both for the probability of infection from the community assuming a constant hazard rate within each time interval, as well as within-school infection during periods where we observed outbreaks as defined above.

S1.2.1.1 Model formulation Let $p_{i,j}$ be the probability of infection of participant i during time interval j defined by time bounds $t \in [t_j^L, t_j^R)$, we have that:

$$
\begin{aligned}
p_{i,j} &= 1 - \exp\left(-\{ \overbrace{\lambda_{C_{k,i}} \Delta t}^{\text{community}} + \overbrace{I(i,j) \lambda_{O_{l,i}}}^{\text{outbreak}} \} \right), \\
\log(\lambda_{C_{k,i}}) &= \alpha_{C_k} + \eta_{C_{k,i}}, \\
\alpha_{C_k} &= \boldsymbol{X}^C_{k.} \boldsymbol{\beta}_C, \\
\eta_{C_{k,i}} &\sim \mathcal{N}(0, \sigma_{C_k}), \\
\log(\lambda_{O_{l,i}}) &= \alpha_{O_l} + \eta_{O_{l,i}}, \\
\alpha_{O_l} &\sim \mathcal{N}(\mu_{\alpha_O}, \sigma_{\alpha_O}), \\
\eta_{O_{l,i}} &\sim \mathcal{N}(0, \sigma_{O_l}),
\end{aligned}
$$

where $\Delta t = t_j^R - t_j^L$ is the length of the time interval, $\lambda_{C_{k,i}}$ is the infection hazard rate parameter from the community during interval j corresponding to conditions $k \in [1, \dots, K]$ that include all K combinations of SARS-CoV-2 variant periods and school vacations represented by covariate matrix $\boldsymbol{X}^C$ and parameter vector $\boldsymbol{\beta_C}$, $I(i,j)$ is an indicator function whether there the participant was in group for which an reported outbreak in interval j, and $\lambda_{O_{l,i}}$ is the infection hazard from outbreak that the participant experience identified by subscript $l \in [1, \dots, 11]$ (see above). Both the community and within-school infection hazards account for individual-level heterogeneity through random effects with event-specific standard deviations σ_C, σ_O.

Having the probability of infection in each time period enables to compute the individual-level probability of past infection at the end of each time interval $t_m = t_m^R$, $\phi_i(t_m)$, using the forward equation:

$$
\begin{aligned}
\phi_i(t_m) &= \phi_i(t_{m-1}) + (1 - \phi_i(t_{m-1}))\, p_{i,m}, \\
\phi_i(t_0) &= p_{0,i}, \\
\text{logit}(p_{0,i}) &\sim \mathcal{N}(\mu_{p_0}, \sigma_{p_0}),
\end{aligned}
$$

where $t_{m-1} = t_{m-1}^R$ corresponds to the right bound of the previous interval, and $p_{0,i}$ is the initial probability of past infection at the beginning of the study period which we model on the logit-scale as having mean μ_{p_0} and standard deviation σ_{p_0}.

S1.2.1.2 Observation likelihoods We have two observation sets that are capillary-blood serologies and RT-PCR and antigen test results, either from our study protocol (in the form of swabs made by the study team of self-reported through questionnaires, see above) or from the state of Geneva's central database of SARS-CoV-2 test results (see above).

S1.2.1.2.1 Serology when antiviral-positive test dates available For serologies we account for both the history of past RT-PCR/antigen test results as well as serology results $y_{i,t}$ through a multinomial distribution. Assuming the date of earliest date of positive RT-PCR/antigen test result t_i^{test} is known for participant i (date of earliest test prior to serology), the likelihood of the serology test result at time t given the probability of past infection $\phi_i(t)$ is given by a multinomial distribution:

$$
\begin{aligned}
\boldsymbol{y^*}_{i,t} &\sim \text{multinomial}([p_1^*, p_2^*]), \\
\boldsymbol{y^*}_{i,t} &= \begin{cases} [0,1] & \text{if} \quad y_{i,t} = 1 \\ [1,0] & \text{if} \quad y_{i,t} = 0 \text{'} \end{cases} \\
p_1^* &= \frac{\theta_{sero}^+(\tau_{i,t})\theta^+\phi_i(t) + (1-\theta_{sero}^-)(1-\theta^-)(1-\phi_i(t))}{\phi(t)\theta^+ + (1-\phi(t))(1-\theta^-)}, \\
p_2^* &= \frac{(1-\theta_{sero}^+(\tau_{i,t}))\theta^+\phi_i(t) + \theta_{sero}^-(1-\theta^-)(1-\phi_i(t))}{\phi(t)\theta^+ + (1-\phi(t))(1-\theta^-)},
\end{aligned}
$$

where $\boldsymbol{y^*}$ is a vector encoding the multinomial draw, p_1^*, p_2^* are the probabilities of positive or negative serology and positive RT-PCR/antigen test respectively accounting for both the sensitivity, θ_{sero}^+, and specificity, θ_{sero}^-, of serology, as well as that of antiviral tests θ^+, θ^-. We accounted for changes in serology sensitivity with time post infection $\tau_{i,t} = t - t_i^{inf}$ as described in the next section.

As described above serology results were produced by an in-house capillary blood test which has been previously validated on clinical samples with reported sensitivity 95.1% of and specificity of 97.6% [6]. However the samples in the validation study were all from adults and around 11 months post-infection. It was therefore uncertain whether these results would transfer to our study population, in particular considering time-varying changes in sensitivity with time post-infection which have been highlighted for other anti-SARS-CoV-2-spike assays [7].

Following Perez-Saez et al. [7] and Azman et al. [8] we therefore modeled time-varying sensitivity using cubic regression on the logit scale:

$$
\text{logit}(\theta_{sero}^+(\tau)) = \gamma_0 + \gamma_1 \log(\tau) + \gamma_2 \log(\tau)^2 + \gamma_3 \log(\tau)^3,
$$

where τ is the time from infection to serology.

S1.2.1.2.2 Serology in the absence of antiviral-positive tests We did not have any antiviral-positive test result for some participants for which we had serologies. In the absence of information on the date of probable infection we marginalized out the unknown date of infection and resulting serology sensitivity following Perez-Saez et al. [7]:

$$
\mathbb{P}(y_{i,t}|\boldsymbol{\Theta}) = \phi_i(t) \times \sum_{j \in \mathcal{S}_{i,t}} \text{Bernoulli}(y_{i,t}|\theta_{sero}^+(t-t_j)))\mathbb{P}(t_j) + (1-\phi_i(t)) \times \text{Bernoulli}(y_{i,t}|1-\theta_{sero}^-),
$$

where $\boldsymbol{\Theta}$ is the vector of all model parameters, Bernoulli is the probability mass function of the Bernoulli distribution, $t_j = t_j^R$ is the right bound of interval j for the set intervals for participant i that precede time

t among all J_i possible intervals $\mathcal{S}_{i,t} := \{k \in [1,\dots,J_i] : t_k^R < t\}$, and $\mathbb{P}(t_j)$ is the relative probability that infection occurred during the interval ending in t_j given that infection occurred by time t.

Given known infection probabilities in each of the intervals leading to time t, the relative probability of earliest infection at time t_j, $\mathbb{P}(t_j)$, was computed as:

$$\mathbb{P}(t_j) = \frac{p_{i,j}\prod_{k<j}(1-p_{i,k})}{\sum_{l\in\mathcal{S}_{i,t}} p_{i,l}\prod_{k<l}(1-p_{i,k})},$$

where $p_{i,j}$ is the probability for participant i to have been infected in time interval j as defined above.

S1.2.1.2.3 Antiviral test results The likelihood of antiviral test results (RT-PCR or antigen) was based on the probability of participant i having been infected in the 7 days prior to the test date t, using the same formulation for the probability of infection as above:

$$\begin{aligned} z_{i,t} &\sim \text{Bernoulli}(\theta^+ p^\star_{i,t} + (1-\theta^-)(1-p^\star_{i,t})),\\ p^\star_{i,t} &= 1 - \exp\left(-\{7 \times \lambda_{C_{k,i}} + I(i,t)\lambda_{O_{l,i}}\}\right), \end{aligned}$$

where here $I(i,t)$ is an indicator function determining whether and outbreak occurred in the 7 days prior to time t in the school group of participant i.

S1.2.1.3 Priors We use the following priors:

$$\begin{aligned} &\text{Community infections :}\\ \beta_C[1] &\sim \mathcal{N}(-1,1),\\ \beta_C[2:K] &\sim \mathcal{N}(0,1),\\ \sigma_C &\sim \mathcal{N}^+(0,2.5),\\ &\text{Outbreak infections :}\\ \mu_{\alpha_O} &\sim \mathcal{N}(-1,1),\\ \sigma_{\alpha_O} &\sim \mathcal{N}^+(0,1),\\ \sigma_O &\sim \mathcal{N}^+(0,2.5),\\ &\text{Initial probability of prior infections :}\\ \mu_{p0_a} &\sim \mathcal{N}(-1.35,.5),\\ \sigma_{p0_a} &\sim \mathcal{N}^+(0,2.5),\\ n_{P_a} &\sim \text{Binomial}(\tilde{p0_a}, N_{general_a}),\\ &\text{Serology :}\\ \gamma_0 &\sim \mathcal{N}(2,.5),\\ \gamma_3 &\sim \mathcal{N}(-1,.5),\\ \text{logit}(\theta^+(1)) &\sim \mathcal{N}(-3,.75),\\ \text{logit}(\theta^+(500)) &\sim \mathcal{N}(0.5,.5),\\ \theta^-_{sero} &\sim Beta(4,1),\\ n_{TP} &\sim \text{Binomial}(\theta^+_{sero}(330), N_{controls+}),\\ n_{FP} &\sim \text{Binomial}(\theta^-_{sero}, N_{controls-}). \end{aligned}$$

The prior for the intercept for community infections, $\beta_C[1]$, corresponds to a weekly infection probability in the range 0.1%-5%, with the priors on the other coefficients $\beta_C[2:K]$, with K the total number of variant and vacation indicator variables, roughly doubling the upper bound of the range for each additional additive parameter.

Priors for outbreak infection hazards were based on published estimates of SARS-CoV-2 outbreak cumulative attack rates in schools in the range of 10%-66% for the Alpha and Omicron BA.1 variants [9, 10]. The choice of priors on $\mu_{\alpha_O}, \sigma_{\alpha_O}, \sigma_O$ results in a prior mean probability of outbreak infection, $\mathbb{E}[p_{outbreak}] = \int_0^1 \text{logit}^{-1}(\alpha_O + \sigma_O \Phi(s))ds$, where Φ is the standard normal quantile function, in the range 5%-75%.

To inform the baseline probability of prior SARS-CoV-2 infection in March 2021 we draw on our previous estimates of population-level seroprevalence by age category in the state of Geneva in December 2020 [3]. Our estimates of seroprevalence ranged from $\approx 15\%$ in among children aged 0-5 to $\approx 25\%$ in adults aged 25-34. Assuming that infection rates were relatively low between the end of the serosurvey and the start of this study, we set priors on the expected probability of baseline infection in each age category a to cover the range of published seroprevalence estimates, $\mathbb{E}[p_{0_a}] = \int_0^1 \text{logit}^{-1}(\mu_{p_{0_a}} + \sigma_{p_{0_a}} \Phi(s))ds$, so that $\mathbb{E}[p_{0_a}]$ lies in the range 12%-45%. We link the age class-specific probabilities of baseline prior infection to the observed number of positives n_{P_a} among those tested in that age class, $N_{general_a}$, in Stringhini et al. [3] through the adjusted seropositivity probability $\tilde{p_{0_a}} = \theta_0^+ \mathbb{E}[p_{0_a}] + (1 - \theta_0^-)\mathbb{E}[p_{0_a}]$ accounting for the sensitivity and specificity of the immunoassay used in that study($\theta_0^+ = 98\%, \theta_0^- = 99\%$).

Priors on the performance of capillary blood serology we choose a beta prior on specificity putting probability mass in higher values (range 40%-100%). As time-varying sensitivity depends on four parameters, we set priors at either end of the range of times post infection (range of 1%-17% at one day post infection and 35%-80% at 500 days post infection), a prior on the intercept γ_0 corresponding to 185 days post-infection for a sensitivity in the range 75%-95%%, and a weak prior on the coefficient multiplying the squared log-time post infection γ_3 favoring a concave shape. The results prior distribution of time-varying serology sensitivity is shown in Figure S4. We further inform the values of sensitivity and specificity with the control data from Michielin et al. [6] where n_{TP}/n_{FP} and $N_{controls+}/N_{controls-}$ are the number of true/false positives and positive/negative controls for capillary blood serology.

S1.2.1.4 Inference We draw inference from the statistical model in a Bayesian framework using Hamiltonian Monte Carlo as implemented in the Stan programming language [11]. We ran four chains of 250 warmup iterations and 1250 sampling iterations and assess convergence through visual inspection of the traceplots and the split R-hat statistic [12].

Model fidelity to observations is given in figure S5.

S1.2.2 Dynamic modeling framework

We do so through a transmission model at the individual level accounting for RT-PCR/antigen test results, serology, and available sequence data.

S1.2.2.1 Transmission model Transmission dynamics are modeled with a compartmental model that tracks individual progression through infection phases within the SIR framework following previous studies on SARS-CoV-2 (Figure S8a) [13]. Susceptible individuals (S), once infected, go through an exposed non-infectious incubation phase (E), followed by two infectious phases subdivided into pre-symptomatic (P) and symptomatic (I). After leaving the infectious period individuals return to a susceptible state with partial protection to subsequent infection (S′). We account for variant-specific natural histories using one E, three P, and one I compartments and variant-specific transition rates between compartments (details in section S1.2.2.3).

We assume that infections can occur either through within-school transmission, or through infections from the community (Figure S8b). We further differentiate within-school transmission between transmission with school classes (*within-group*) or between classes (*between-group*). To account for participants living in the same household but attending different classes, we further account for household-level transmission (*hh*). Following Cauchemez et al. [14], let $\lambda_{i,t}$ denote the instantaneous hazard rate experienced by participant i at time t. This hazard is composed of two parts, the hazard from all potentially infectious contacts and the hazard from the community:

$$\lambda_i = \sum_{j \in \mathcal{S}_i} \beta_{i,j} I^\star_j + \beta_C,$$

where $\mathcal{S}_i$ is the set of all individuals in contact with i (either in same household, same class, or same school), $I^\star_j$ denotes whether individual j is in one of infectious states $\{P_1, P_2, P_3, I\}$, β_C is the community infectious pressure, and $\beta_{i,j}$ is the hazard coefficient represents the contact type between i and j. We differentiate between the three types of possible contacts:

$$\beta_{i,j} = \begin{cases} \beta_h & \text{if same household,} \\ \beta_g & \text{else if same class,} \\ \beta_s & \text{else if same school,} \\ 0 & \text{otherwise.} \end{cases}$$

As a first pass we set $\beta_h = \beta_g$ for simplicity. We here assume that transmission does not vary with respect to the age of infector-infectee pairs. This is support by evidence from all three Alpha, Delta and Omicron variants [15, 16]. We here therefore aim at inferring the parameter set $\Theta = \{\beta_g, \beta_s, \beta_C\}$ for each SARS-CoV-2 variant.

S1.2.2.2 Stochastic model implementation To account for the discrete nature of individual-level transition between states we implement the model as a continuous time Markov process, or more precisely as a stochastic counting process [17]. We follow the convention of denoting $N_{AB}(t)$ as the number of individuals transitioning between classes ($A, B \in \mathcal{X}$) in the time interval $[0, t)$, where $\mathcal{X} = \{S, E, P_{1-3}, I, S'\}$ is the set of possible states an individual can be in, and $N_{\bullet A}$ denote the number of "births" into A and $N_{A\bullet}$ the number of exits from A. The number of transition during a timestep Δt is $\Delta N_{AB}(t) = N_{AB}(t + \Delta t) - N_{AB}(t)$, which in our specific case can only take the values of 0 or 1 as we model each individual separately. Given the state of the system at time t, $\mathcal{X}_t$, the transition rates read:

$$\begin{aligned} \mathbb{P}\left[\Delta N_{SE_i}(t) = 1 | \mathcal{X}_t\right] &= \lambda_{i,t} S(t)\Delta t + o(\Delta t) \\ \mathbb{P}\left[\Delta N_{S'E_i}(t) = 1 | \mathcal{X}_t\right] &= \eta\lambda_{i,t} S(t)\Delta t + o(\Delta t) \\ \mathbb{P}\left[\Delta N_{EP_{1i}}(t) = 1 | \mathcal{X}_t\right] &= \phi E(t)\Delta t + o(\Delta t) \\ \mathbb{P}\left[\Delta N_{P_1 P_{2i}}(t) = 1 | \mathcal{X}_t\right] &= \phi P_1(t)\Delta t + o(\Delta t) \\ \mathbb{P}\left[\Delta N_{P_2 P_{3i}}(t) = 1 | \mathcal{X}_t\right] &= \phi P_2(t)\Delta t + o(\Delta t) \\ \mathbb{P}\left[\Delta N_{P_3 I_i}(t) = 1 | \mathcal{X}_t\right] &= \phi P_3(t)\Delta t + o(\Delta t) \\ \mathbb{P}\left[\Delta N_{P_3 I_i}(t) = 1 | \mathcal{X}_t\right] &= \phi P_3(t)\Delta t + o(\Delta t) \\ \mathbb{P}\left[\Delta N_{IS'_i}(t) = 1 | \mathcal{X}_t\right] &= \gamma I(t)\Delta t + o(\Delta t) \end{aligned}$$

where λ_i is the hazard rate experienced by individual i as described above, $\eta \in [0, 1]$ is a parameter representing reduced susceptibility following infection or vaccination (see details in section S1.2.2.4), ϕ is the transition rate between the exposed and pre-symptomatic compartments (see details in section S1.2.2.3), and γ is the transition rate out of the symptomatic and infectious compartment. We note that all parameters were made variant-specific. To complete the model definition, we further assume that $\mathbb{P}[\Delta N_{AB} > 1 | \mathcal{X}_t] = 0 \; \forall A, B \in \mathcal{X}$ and $\mathbb{P}[\Delta N_{A\bullet} > 1 | \mathcal{X}_t] = 0 \; \forall A \in \mathcal{X}$.

The resulting stochastic variations of the state variables are:

$$
\begin{aligned}
\Delta S_i(t) &= -\Delta N_{EI_i}(t), \\
\Delta E_i(t) &= \Delta N_{SE_i}(t) + \Delta N_{S'E_i}(t) - \Delta N_{EP_1}(t), \\
\Delta P_{1_i}(t) &= \Delta N_{EP_{1_i}}(t) - \Delta N_{P_1P_{2_i}}(t), \\
\Delta P_{2_i}(t) &= \Delta N_{P_1P_{2_i}}(t) - \Delta N_{P_2P_{3_i}}(t), \\
\Delta P_{3_i}(t) &= \Delta N_{P_2P_{3_i}}(t) - \Delta N_{P_3I_i}(t), \\
\Delta I_i(t) &= \Delta N_{P_3I_i}(t) - \Delta N_{IS'_i}(t), \\
\Delta S'_i(t) &= \Delta N_{IS'_i}(t) - \Delta N_{S'E_i}(t).
\end{aligned}
$$

S1.2.2.3 SARS-CoV-2 variants

S1.2.2.3.1 Mechanistic modeling of infection stages We draw on published estimates of the natural history parameters for the Alpha, Delta and Omicron BA.1 SARS-CoV-2 variants. We follow the mechanistic approach proposed in Hart et al. [13] which explicitly accounts for the pre-symptomatic stage in linking incubation period and generation times, thus mapping to the model diagram in section S1.2.2.1. The model assumes that the time an individual stays in each E, P, and I stages follow a Gamma distribution. Using Hart et al. [13]'s notation, the incubation period is also assumed to follows a Gamma distribution with shape parameter k_{inc} and scale parameter $\rho_{inc} = 1/(k_{inc}\gamma)$, where γ is the mean incubation time. The different stages can then be linked by assuming that the incubation period can be partitioned between the E and P stages with equal scale parameters, and that the symptomatic infectious stage I has its own shape and scale parameters:

$$
\begin{aligned}
y_{inc} &\sim \text{Gamma}(k_{inc}, \rho_{inc}), \\
y_E &\sim \text{Gamma}(k_E, \rho_{inc}), \\
y_P &\sim \text{Gamma}(k_P, \rho_{inc}), \\
y_I &\sim \text{Gamma}(k_I, \rho_I), \\
k_{inc} &= k_E + k_P,
\end{aligned}
$$

where $y_{inc/E/P/I}$ denotes the gamma-distributed random variable of time spent in each stage, and $\rho_I = 1/(k_I\mu)$ is the scale parameter of the symptomatic infectious stage resulting in a mean symptomatic period of μ.

As a result of this formulation one can derive a close-form solution for the mean and variance of the intrinsic generation time distribution (in the absence of susceptible depletion), y^* [13]:

$$
\begin{aligned}
\mathbb{E}[y^*] &= C_1(\alpha_P\mathbb{E}[y_P^2] + 2\mathbb{E}[y_P]\mathbb{E}[y_I] + \mathbb{E}[y_I^2]) \\
\mathbb{V}[y^*] &= C_2(\alpha_P\mathbb{E}[y_P^3] + 3\mathbb{E}[y_P^2]\mathbb{E}[y_I] + 3\mathbb{E}[y_P]\mathbb{E}[y_I^2] + \mathbb{E}[y_I^3]) - \mathbb{E}[y^*]^2,
\end{aligned}
$$

where α_P is a multiplicative factor linking pre-symptomatic and symptomatic infectiousness, and $C_{1/2}$ are normalization constants with closed-form expressions. This modeling framework allowed Hart et al. [13] to infer the incubation period and generation times for the Alpha and Delta variants from household data on which we based the parametrization of our transmission model.

S1.2.2.3.2 Parametrization of compartment residence times Our model is implemented in the form of a compartmental model with discrete transitions, which by construction imposes an exponentially distributed residence time in each stage. We can however represent Erlang-distributed, ie. Gamma distribution with integer shape parameter, residence times by concatenating compartments of the same type, as originally done in King et al. [18] and implemented in Lemaitre et al. [19] for SARS-CoV-2. Published estimates of the incubation period and generation time distributions by variant can then be used to find the parametrization of the compartmental model that best reproduces the inferred time distributions. For each variant v, we are therefore interested in finding the set of values $\theta_v = \{k_{E,v}, k_{P,v}, k_{I,v}, \rho_{I,v}\}$ that best match inferred estimates

of the incubation period $f_{inc}(y|k_{inc}, \rho_{inc})$ and of the mean and variance of the generation time $\mathbb{E}[y^*]$, $\mathbb{V}[y^*]$. To this end we implement the follow the following approach:

1. Find the best value of $k'_{inc} \in \mathbb{Z}^+, \rho'_{inc} \in \mathbb{R}+$ that minimize the Kullback-Leibler divergence with $f_{inc}(y|k_{inc}, \rho_{inc})$.
2. Using k'_{inc}, ρ'_{inc}, find the values of $k_I \in \mathbb{Z}^+, \rho'_I \in \mathbb{R}+$ that minimize the squared distance with $\mathbb{E}[y^*], \mathbb{V}[y^*]$.

The resulting optimal parameters are illustrated in Figure S9.

S1.2.2.3.3 Cross-variant protection from re-infection We use data from the systematic review in Stein et al. [20] to model the cross-variant protection from re-infection. To do so we assume that individuals in compartment S' experience a force of infection $c_{v,u}\lambda_i$ where $c_{v,u}$ is the estimate of protection efficacy conferred by previous infection by SARS-CoV-2 variant v on re-infection by variant u.

S1.2.2.4 Vaccination A number of school staff and 3 children were vaccinated during the study period (Table S1-S2). As anti-SARS-CoV-2 vaccines only provide partial protection against infection, we did not model a separate compartment for vaccination, and instead assumed that vaccinees are transferred to the S' compartment which already accounts for partial protection. Vaccine-induced protection was reported to be of similar magnitude than previous infection by the ancestral SARS-CoV-2 variant and therefore used the same values for both [20].

S1.2.2.5 Contact matrices and vacations We account for the effect of school-year vacations on school-based transmission by modifying the contact matrix between participants and turning off within-school contacts during vacation periods. We implement this change during the official vacations dates in the state of Geneva.

S1.2.2.6 Individual and bubble quarantine protocols Public health measures to curb SARS-CoV-2 transmission in the state of Geneva included mandatory quarantine following positive anti-viral test results, as well as targeted bubble quarantines in schools with outbreaks. The protocols for both measure changed during the study period, in particular the length of quarantine which was of 7 days prior to January 12th 2022, and 5 days from January 13th onward. We implemented both measures in the transmission model by modifying the contact matrix based on reported positive anti-viral tests as well as bubble quarantines notified by the schools. In the model implementation we assumed that individuals conform to the quarantine lengths.

S1.2.2.7 Observation likelihoods At any time point we may have three sources of data for a study participant: anti-viral (RT-PCR/antigen) test results, capillary-blood serology, and WGS. For the latter two we follow a similar approach as the statistical model, and for the latter we adapt methods from genetic-based methods for inferring transmission chains.

S1.2.2.7.1 Anti-viral tests Knowing the state of participant i at time t, $\mathcal{C}_{i,t}$, we assume that the anti-viral test result, $z_{i,t}$, follows a Bernoulli distribution:

$$\begin{aligned} z_{i,t} &\sim \text{Bernoulli}(\theta^+ x_{i,t} + (1-\theta^-)(1-x_{i,t})), \\ x_{i,t} &= I(\mathcal{C}_{i,t} \in \{P_1, P_2, P_3, I\}), \end{aligned}$$

where θ^+, θ^- are the test's sensitivity and specificity, and $I(\mathcal{C}_{i,t} \in \{P_1, P_2, P_3, I\})$ is an indicator variable denoting whether i is in an infectious state or not.

S1.2.2.7.2 Serology Similarly, we assume that capillary-blood test result, $y_{i,t}$, follows a Bernoulli distribution:

$$y_{i,t} \sim \text{Bernoulli}(\theta^+_{sero}(t - Y_i)x'_{i,t} + (1-\theta^-_{sero})(1-x'_{i,t})),$$

where the sensitivity θ^{+}_{sero} is assumed to vary with time from infection as inferred in the statistical modeling framework (see section S1.8.1.2.1), Y_i is the time of earliest infection of participant i, $x'_{i,t} \in 0, 1$ is a binary variable indicating whether participant i has experienced infection or vaccination in the by time t, and θ^{-}_{sero} is the estimated test specificity.

S1.2.2.7.3 Genetic likelihood To incorporate the WGS data we collected during outbreak investigation we followed the approach proposed by Jombart et al. [21] for the reconstruction of transmission chains based on genetic data. As described in the next section we adopt a simulation-based inference method that enjoys the plug-and-play characteristic [17], which is why we speak about simulated states but in the context of parameter inference.

We start by defining the likelihood of the number of base differences between sequences q and r, $\delta_{q,r}$, that are assumed to be separated by a known number $\kappa \in \{1, \ldots, \kappa_{max}\}$ infection generations apart, and share a common sequenced sequence length $L_{q,r}$, assuming a mutation rate ξ as:

$$\delta_{q,r} \sim \text{Binomial}(\kappa\xi, L_{q,r}),$$

where it is assumed that no reverse-mutations can occur. In the framework of Jombart et al. [21] inference focuses on infection cases for which a sequence is available, and the method treats infection times and infector-infectee pairs as parameters to draw posterior samples from. In this study we are simulating explicitly the infection status of each individual which therefore enables to explicit use the infection states of all individuals to determine the genetic likelihoods. Based on the description of the phylogenetic inference described above, we can distinguish three cases of sequence data and simulated states:

No sequence ancestors The sequence is at the root of the photogenic tree of study sequences for the given period, and it can only be linked to community sequences. If we assume that the mutation rate ξ is known, the likelihood for these sequences does not depend on the rest of the model components. It is therefore a constant and can be ignored.

No potential infectors The simulation at time t_q at which sequence q was sampled does not predict any infectious individuals in contact with individual i_q from which the sequence was sampled from. The genetic likelihood of this sequence can therefore only be linked to community sequences. Let s_{q^C} be the closest community sequence based on the maximum likelihood tree, and t_{q^C} it's sampling time. Following Jombart et al. [21], the likelihood of s_q requires the marginalization of the unknown number of infection generations separating it form s_{q^C} :

$$\mathcal{L}(s_q|\xi, s_{q^C}, t_q, t_{q^C}) = \sum_{\kappa=1}^{\kappa_{max}} \text{Binomial}(\delta_{q,q^C}|\kappa\xi, L_{q,q^C})\mathbb{P}(\kappa|t_q, t_{q^C}),$$
$$\mathbb{P}(\kappa|t_q, t_{q^C}) = f^{\kappa}_{gen}(t_{q^C} - t_q),$$

where $\mathbb{P}(\kappa|t_q, t_{q^C})$ is the probability of sequence sampling times t_q and t_{q^C} to be separated by κ infection generations in terms of the generation interval pdf, f_{gen}, convoluted κ times, $f^{\kappa}_{gen} = f_{gen}\underbrace{*\cdots*}_{\kappa}f_{gen}$. For simplicity we take the Alpha estimate of the generation interval in Hart et al. [13] parametrized as a gamma distribution $f_{gen} = \text{Gamma}(1.89, 2.91)$.

Potential infectors When the school-based force of infection the individual experiences at time t_q is non-null, meaning that sequence could be linked to previous sequences through a transmission chain, we need to marginalize out possible within-school and community transmission pathways. Let $\mathcal{A}_q$ be the set of possible sequences that are ancestors of q based on the maximum likelihood tree, corresponding to the set $\mathcal{I}_q$ of individuals that have experienced an infection in the past within the simulation, we have that the likelihood of sequence s_q is composed of the probability of within-school transmission and that of community importation as:

$$\mathcal{L}(s_q) = \overbrace{\sum_{\{j,r\}\in\{\mathcal{I}_q,\mathcal{A}_q\}} \mathcal{L}(s_q|s_r,x_j)\mathbb{P}(j)}^{\text{within-school}} + \overbrace{\mathbb{P}(C)\sum_{\kappa=1}^{\kappa_{max}} f(q,q^C,\kappa)\mathbb{P}(\kappa|t_q,t_{q^C})}^{\text{community}},$$

$$\mathcal{L}(s_q|s_r,x_j) = \begin{cases} f(q,r,1) & \text{if} \quad x_j = 1 \\ \sum_{\kappa=2}^{\kappa_{max}} f(q,r,\kappa)\mathbb{P}(\kappa|t_q,t_r) & \text{if} \quad x_j = 0 \end{cases},$$

$$\mathbb{P}(j) = \begin{cases} \frac{\beta_{i,j}}{\lambda_i^{S\star}+\beta_C} & \text{if} \quad x_j = 1 \\ \frac{(\lambda_i^{S\star}-\beta_i^\star)/n_i^\star}{\lambda_i^{S\star}+\beta_C} & \text{if} \quad x_j = 0 \end{cases},$$

$$\mathbb{P}(C) = \frac{\beta_C}{\lambda_i^{S\star}+\beta_C},$$

where $f(q,r,\kappa) = \text{Binomial}(\delta_{q,r}|\kappa\xi, L_{q,r})\mathbb{P}(\kappa|t_q,t_r)$ is a shorthand for the likelihood of base differences as defined above, $\lambda_i^{S\star}$ is the school-based hazard from the $n_i^\star$ individuals that are potential ancestors for i, i.e. all $j \in \mathcal{I}_i$ that were in an infectious state at time t_q, and $\beta_i^\star = \sum_{k\in\mathcal{I}_i} I(k)\beta_{i,k}$ is the sum of the hazard rates from those individuals with $I()$ an indicator function of whether the individual k is infectious or not. By design the sum of the within school and community marginal probabilities sum to one: $\mathbb{P}(C) + \sum \mathbb{P}(i) = 1$.

S1.2.2.7.4 General population seroprevalence data The school-based investigation in this study was conducted in parallel to general-population seroprevalence studies in the state of Geneva by our group throughout the different phases of the pandemic [3, 4, 5]. We therefore incorporate seroprevalence estimates in our modeling analysis to inform cumulative attack rate trajectories. We do so by linking class-level cumulative infection rates to estimated seroprevalence including.

The serosurvey of November/December 2020 in Stringhini et al. [3] was used to inform the initial infection conditions as:

$$C_{t_0,g,a} \sim \text{Binomial}(N_{g,a}, \hat{p}_{1,a}),$$

where $C_{t_0,g,a}$ is the number of previously exposed individuals at the start of the simulation in school group g and age class a, $N_{g,a}$ is the total number of individuals in that group and age class, and $\hat{p}_{1,a}$ is the estimated seroprevalence in age group a in December 2020.

Similarly we link the serosurvey of June/July 2021 in Stringhini et al. [4] to the simulated state of infections at that date:

$$C_{t_1,g,a} \sim \text{Binomial}(N_{g,a}, \hat{p}_{2,a}),$$

where t_1 is the time of serosuervey end (July 7 2021), and $\hat{p}_{2,a}$ the corresponding seroprevalence estimate. Note that due to access to vaccines during 2021 [4], seroprevalence accounts both for infection- and vaccine-induced antibodies. This is aligned with compartment C that accounts both for infection and vaccinations, as the capillary blood serology in this study targeted anti-spike SARS-CoV-2 antibodies [22].

Our school-based analysis ends in February 25th 2022, and the last serosurvey was done in June/July 2022, with successive infection waves in between due to the rise of Omicron BA.2 and Omicron 4/5 waves [5]. We therefore could not consider that the state of the system at the end of the modeling period was representative of cumulative attack rates at the serosurvey period. We therefore consider cumulative infections/vaccinations as censored observations of the final cumulative exposure size as:

$$\begin{aligned} \mathcal{L}(C_{t_T,g,a}) &= \mathbb{P}(X \geq C_{t_T,g,a}|N_{g,a}, \hat{p}_{3,a}), \\ &= \text{Binomial}(C_{t_T,g,a}|N_{g,a}, \hat{p}_{3,a}) + \text{BinomialCCDF}(C_{t_T,g,a}|N_{g,a}, \hat{p}_{3,a}), \end{aligned}$$

where t_T is the end of our analysis (Feb 25th 2022), $\hat{p}_3$ is the estimated seroprevalence of anti-spike SARS-CoV-2 antibodies from any origin in the third seroprevalence round in Zaballa et al. [5], and $BinomialCCDF$ is the complementary CDF of the Binomial distribution ($\mathbb{P}(X > x)$).

We marginalize out uncertainty in the estimates of $\hat{p}_1, \hat{p}_2, \hat{p}_3$ by taking the average of observation likelihoods over 100 draws of their posterior distribution.

S1.2.2.8 Inference We draw inference from the dynamical modeling framework through a recently proposed iterated block particle filtering algorithm allowing for dynamical coupling between units [23, 24]. The approach builds on previous successes in inference through iterated particle filters that enjoy the plug-and-play property, meaning that they are simulation-based and gradient-free [25]. The development of block particle filtering (BPF) allows the extension of this approach to systems with coupled dynamics between units [24], and has been illustrated on real-world disease meta-population problems [23].

We here use the BPF implementation from the `spatPomp` R package in Asfaw et al. [26] to perform maximum-likelihood inference. To do so, we perform 100 iterated BPF iterations with 2,500 particles, and evaluate the log-likelihood over 5 replications of the BPF on the final parameter set using 5,000 particles. After an initial parameter search using 35 random initial starting points, we produce parameter profiles over relevant parameter ranges using 9 equally spaced points and five parameter search replications with random starting points. We then use parameter profiles to estimate Monte Carlo parameter confidence intervals following Ionides et al. [27].

Fixed and inferred model parameters are given in Table S8.

Model fidelity to data is shown in figure S10.

S1.3 Scenario simulations

S1.3.1 Main analysis

We use the dynamic model to investigate the effect of community-acquired infections on the effectiveness of within-school interventions. To do so we simulate intervention scenarios considering a reduction in the within-class, or between-class transmission rates as:

$$\beta'_g = \eta_g \beta_g,$$
$$\beta'_s = \eta_s \beta_s,$$

where β_g, β_s are the MLE within-class and between class transmission parameters, and $\eta_g, \eta_s \in [0, 1]$ are the reduction coefficients. We explore reduction coefficients between 0 (transmission interruption) and 1 (baseline scenario of no change in transmission), yielding 121 parameters combinations of $\{\eta_g, \eta_s\}$ in a 0.1 resolution grid ranging from 0 to 1.

For each intervention scenario, we ran simulations for the three epidemiologic settings based on our VOC-specific inference results: very rare community introductions (Alpha-like), rare introductions (Delta-like), and frequent introductions (Omicron-like). For each epidemiologic setting and intervention scenario we ran 1,000 simulation of the dynamic model for a duration of 90 days.

S1.3.2 Sensitivity analyses

S1.3.2.1 Feedback between within-school interventions and community transmission Reduction in within-school transmission may have a feedback on reducing household and community-wide transmission, as highlighted during the COVID-19 pandemic [28, 29]. In sensitivity analysis we consider the impact of this feedback effect on the outcomes of outcomes of scenarios of within-school interventions. Empirical estimates suggest that interruption of within-school transmission during school closures lead to a reduction of 20 to 50 % of community-wide transmission rates [30, 31]. We use these estimates to set the maximum of the potential feedback effect of within-school interventions, and then assume a linear relation between the reduction in community-acquired transmission, η_C, and the within- and between-school intervention reductions as:

$$\eta_C = 1 - 0.5 \times (\frac{1-\eta_g}{2} + \frac{1-\eta_s}{2}),$$

where the maximal effect is of $\eta_C = 50\%$ reduction for total interruption of within-school transmission ($\eta_g = 0; \eta_s = 0$), and no reduction ($\eta_C = 1$) when no within-school interventions are applied ($\eta_g = 0; \eta_s = 0$). Sensitivity results are shown in Figure S13.

S1.3.2.2 Simulation duration In addition to the 90-days simulations in the main, we also considered simulation durations of 180 and 365 days (Figure S14).

S1.4 Data on school closure timings during the COVID-19 pandemic

We assess the timing of school closures during the COVID-19 with respect to trends in reported incidence. We download global data on school closures [32], rolling 7-day mean reported incidence [33], and estimated effective reproduction numbers [33], made available through the Our World in Data portal [34].

As SARS-CoV-2 testing rates and underlying incidence changed importantly during the COVID-19 we compared the timing of school closures by computing the yearly-standardized 7-day mean incidence: for each calendar year and each country we standardize the rolling 7-day mean reported incidence taking the mean and sd of that year. We then produce scatterplots of the yearly-standardized incidence vs. the value of the effective reproduction number on the date of the school closure (Figure **??**). We define high incidence as school closure dates on which the yearly-standardized 7-day mean incidence was larger or equal to 1, and the effective reproduction number was above one.

S2 Supplementary Figures

S2.1 Figure S1: Study flow chart

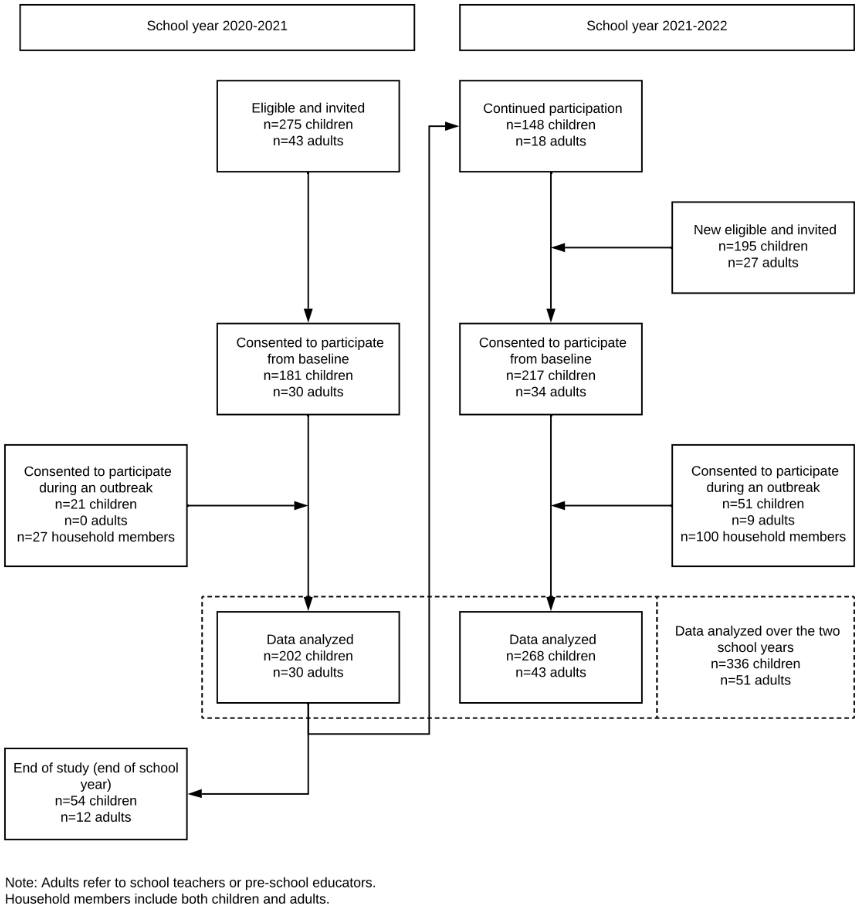

Figure S1: Flow chart.

S2.2 Figure S2: Stringency of non-pharmaceutical interventions in Switzerland

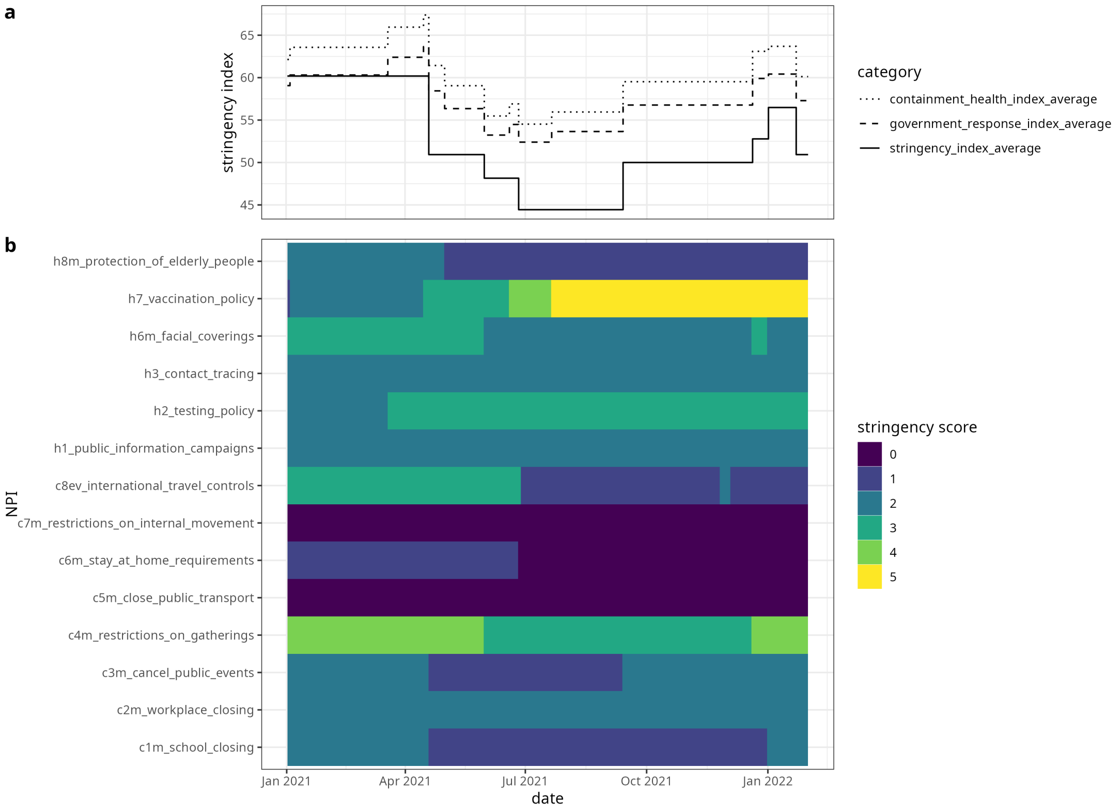

Figure S2: Stringency of non-pharmaceutical interventions (NPIs) in Switzerland during the study period. NPI stringency data from the Oxford Covid-19 Government Response Tracker [32]. a) Average stringency indices by category and overall. b) Stringency scores by NPI.

S2.3 Figure S3: Longitudinal participant data

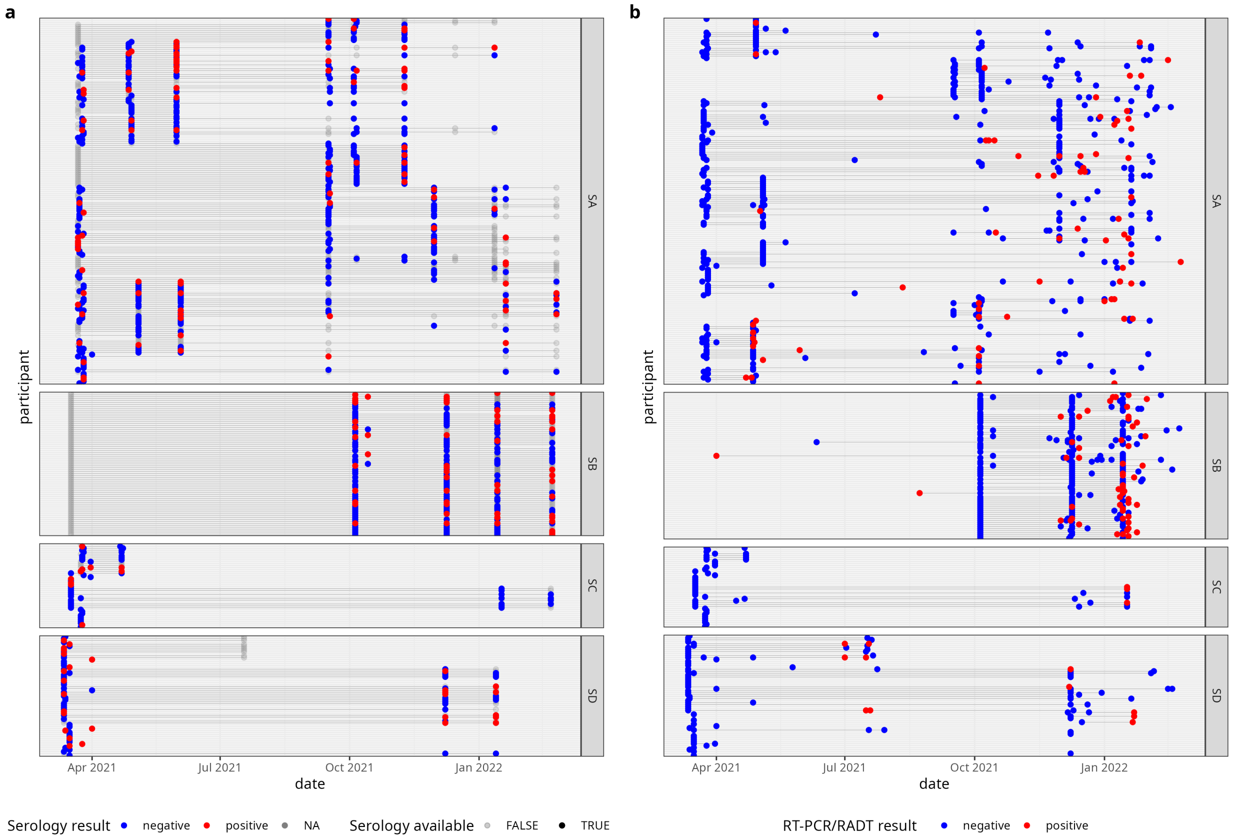

Figure S3: Longitudinal data produced in the SeroCoV-Schools study. Each line represents a participant, points indicate sampling dates. a) Serology test results. All serologies were collected within the SeroCoV-Schools study protocol, either at baseline our during outbreak visits. b) RT-PCR/RADT test results. Results are available either from the SeroCoV-Schools study, or from the Republic and Canton of Geneva's Directorate of Health central repository of anti-SARS-CoV-2 tests made in the canton.

S2.4 Figure S4: Sequence alignments

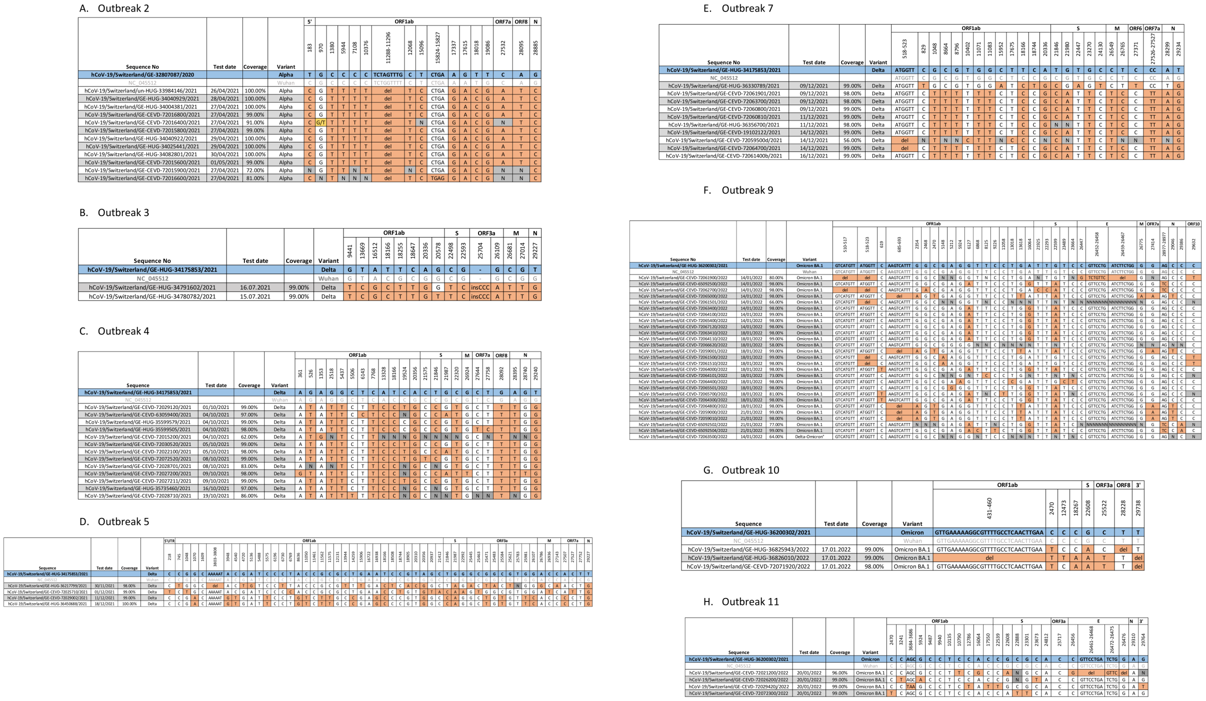

Figure S4: Alignment of SARS-CoV-2 sequences of the positive cases classified by outbreak. As VOC reference sequences, we used the first sequence of each variant (Alpha, Delta and Omicron BA.1) obtained in Geneva, i.e, hCoV-19/Switzerland/GE-32807087/2020, hCoV-19/Switzerland/GE-HUG-34175853/2021 and hCoV-19/Switzerland/GE-HUG-36200302/2021. NC-045512 is a pre-VOC reference sequence. Mutations in comparison to the VOC reference sequences are highlighted in orange. White fields indicate no mutation; grey fields indicate insufficient genome coverage; and yellow fields indicate mixed viral population of the two nucleotides given. Numbers indicate nucleotide position; letters the nucleotide present at this position. del: deletion; ins: insertion; GISAID: Global Initiative on Sharing Avian Influenza Data.

S2.5 Figure S5: Statistical model fidelity to data

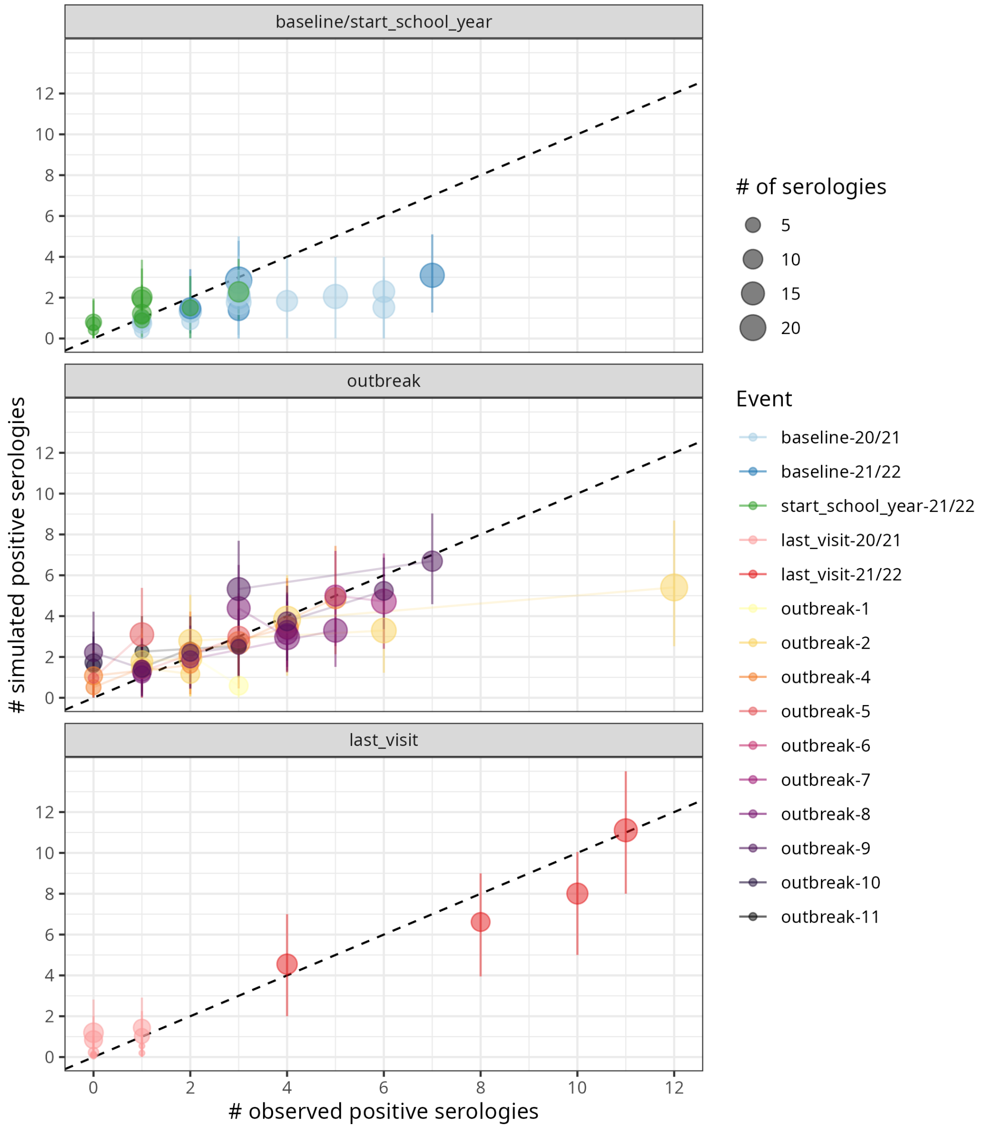

Figure S5: Observed positive serological samples against posterior predictions from the statistical model. Results are grouped by event (baseline, school start, outbreak) and school group. Point size indicates the number of serological samples taken on the event in each school group. Dots indicate the mean and bars the 95% CrI of the posterior distribution based on 5000 HMC draws. Dotted lines indicate the 1:1 ratio representing perfect model fidelity to data.

S2.6 Figure S6: Inference of time-varying serology sensitivity

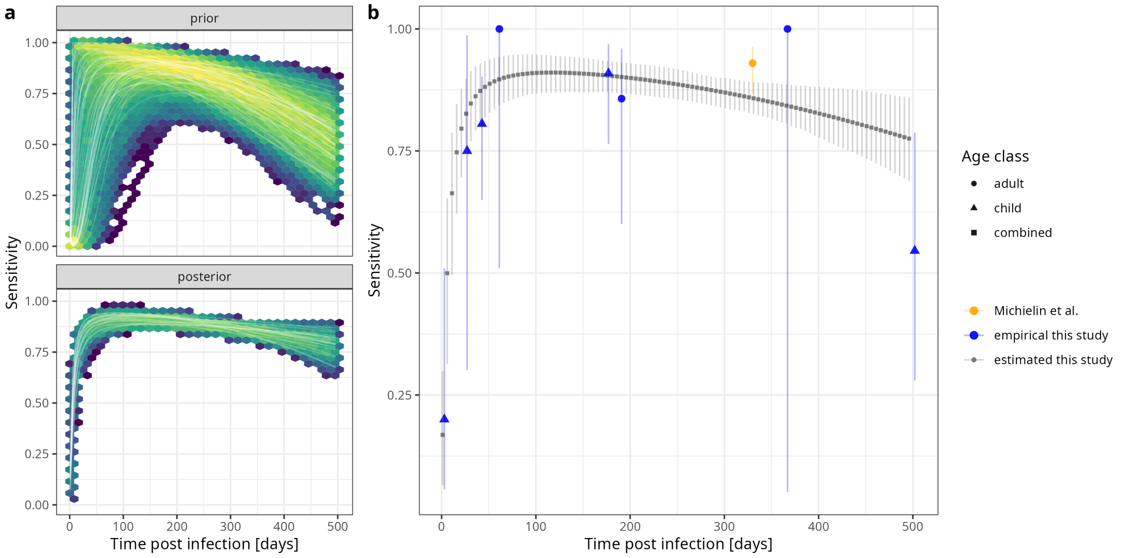

Figure S6: Inference of time-varying capillary-blood serology sensitivity. a) Prior (top) and posterior (bottom) draws of trajectories of sensitivity with time post infection. Colors indicate density of trajectories in each hexbin, and white lines show 50 trajectory draws. b) Comparison of estimated sensitivity (gray squares indicate the mean, bars give 95% credible intervals based on 5000 HMC draws) against empirical estimates within the study sample for participants for which a positive anti-viral test result was available followed by subsequent serology tests (blue dots), and against data published in Michielin et al. (2023) (yellow dots). Empirical data are shown by age class (adults vs. children), and bars give 95% Wilson binomial confidence intervals.

S2.7 Figure S7: Statistical modeling inference of outbreak attack rates

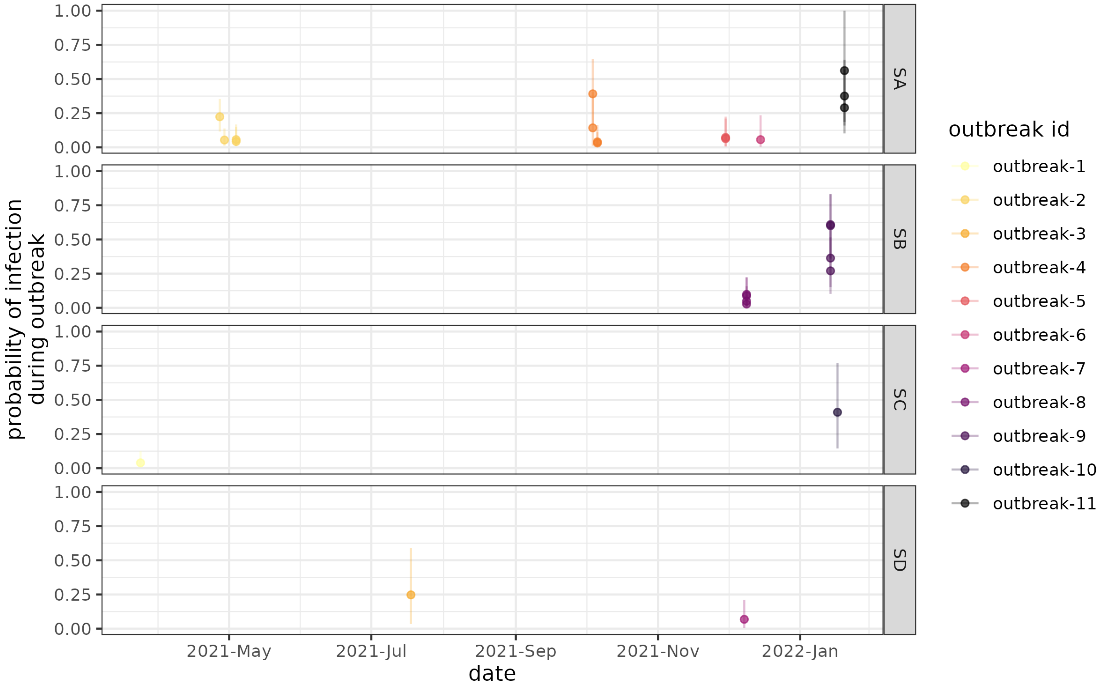

Figure S7: Inferred outbreak attack rates from statistical model. The probability of infection during an outbreak is given by school group (dots:mean, bars: 95%CrIs based on 5000 HMC draws) colored by outbreak number.

S2.8 Figure S8: School SARS-CoV-2 transmission model diagram

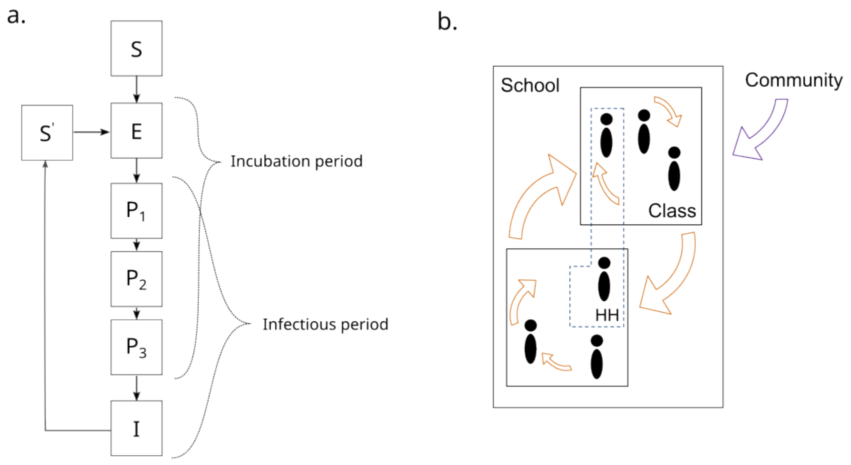

Figure S8: Transmission model. a) Individual-level compartmental model of infection progression. b) Schematic of transmission process.

S2.9 Figure S9: Natural history parameters

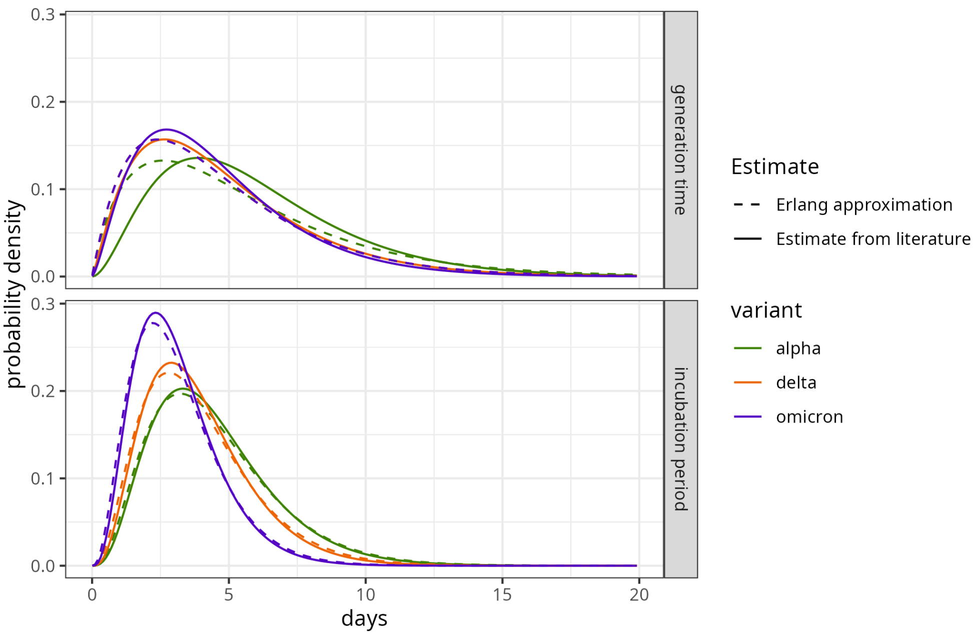

Figure S9: Generation time and incubation periods used in epidemic model.

S2.10 Figure S10: Dynamic model fidelity to data

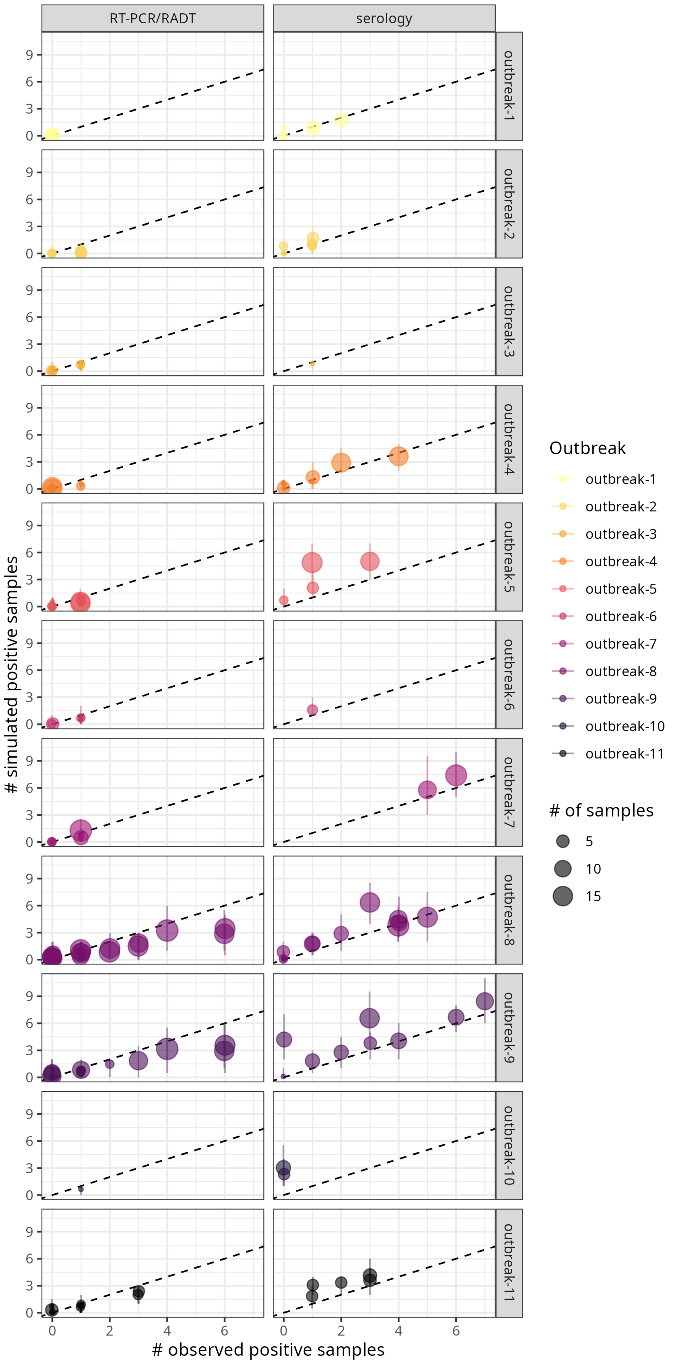

Figure S10: Observed vs. predictions of positive RT-PCR/RADT and serologies from the dynamical model. Results are grouped by outbreak, sampling date and school group. Point size indicates the number of samples taken on the event in each school group. Dots indicate the mean and bars the 95% CI of the smoothing distribution based on 1000 particles. Dotted lines indicate the 1:1 ratio representing perfect model fidelity to data.

S2.11 Figure S11: Comparison of model predictions

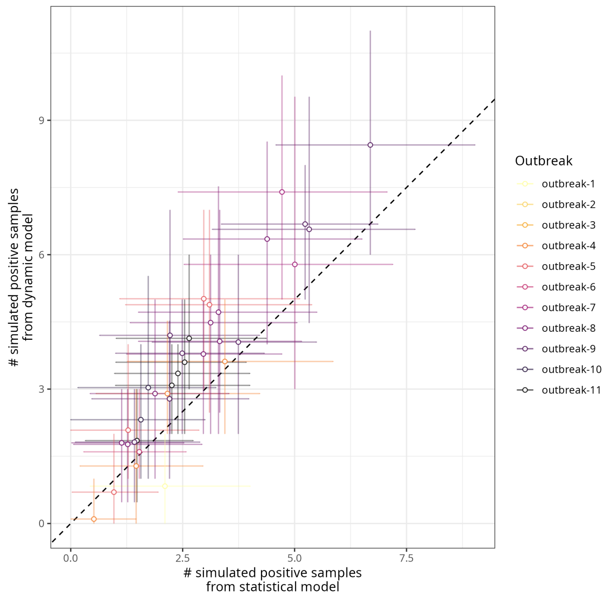

Figure S11: Comparison of predicted positive serologies from the statistical and dynamical models. Results are grouped by outbreak, sampling date and school group. Dots indicate the mean, horizontal bars the 95% CrI of the posterior distribution based on 5000 HMC draws and vertical bars of the smoothing distribution based on 1000 particles, vertical bars the 95% CI of the smoothing distribution based on 1000 particles. Dotted lines indicate the 1:1 ratio representing perfect model fidelity to data.

S2.12 Figure S12: Scenario simulations sensitivity all results

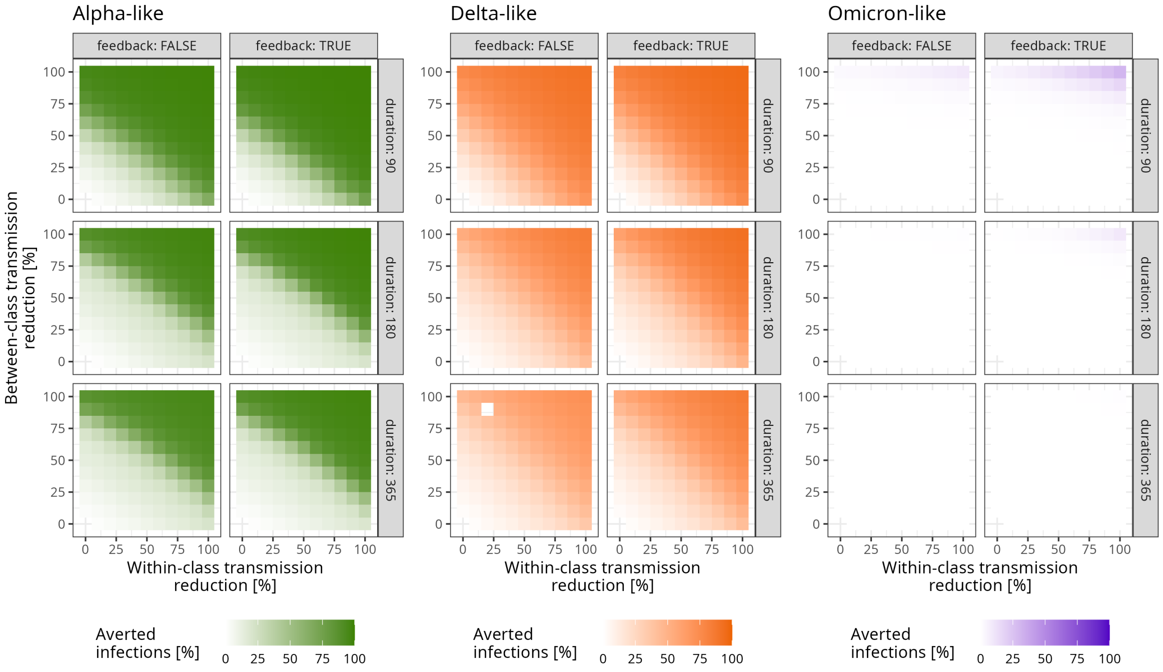

Figure S12: Sensitivity analysis of scenario simulations. Legend as in Figure 4a of the main text. Sensitivity analysis was performed by varying the duration of simulations (90, 180 and 265 days), and accounting for feedback between the reduction of within-school and community transmission (TRUE: feedback account, FALSE: no feedback).

S2.13 Figure S13: Scenario simulations sensitivity by feedback

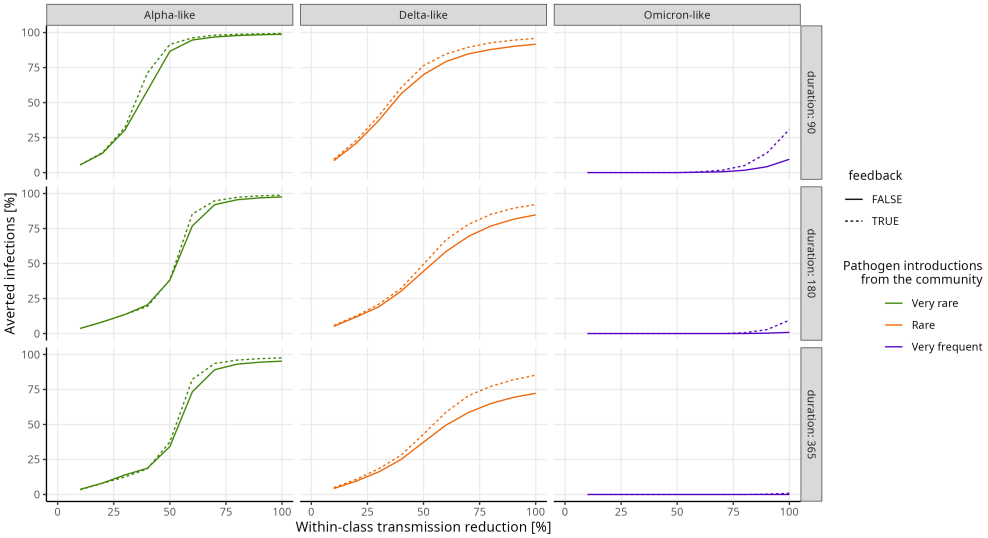

Figure S13: Sensitivity analysis of scenario simulations along the diagonal of scenario space by whether feedback was accounted for or not. Legend as in Figure 4b of the main text.

S2.14 Figure S14: Scenario simulations sensitivity by duration

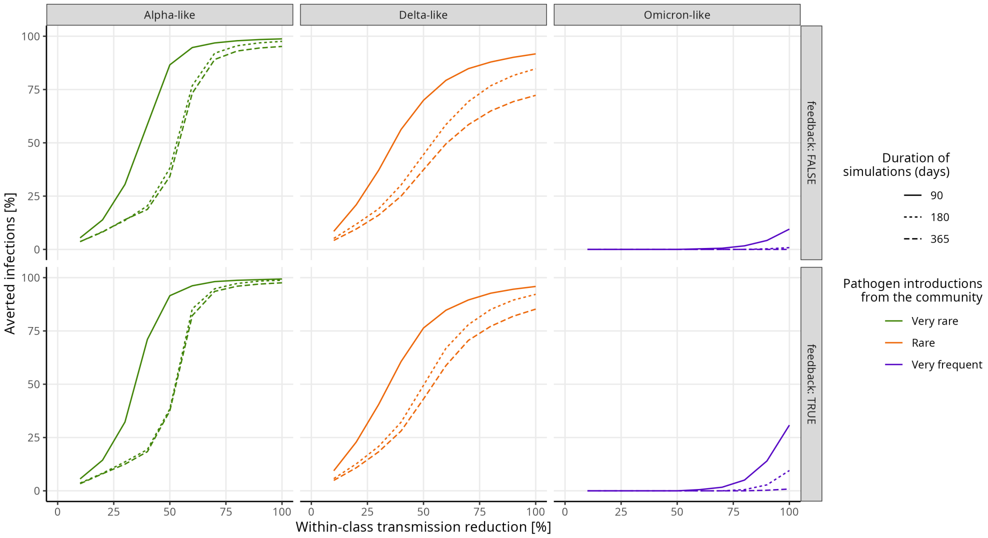

Figure S14: Sensitivity analysis of scenario simulations along the diagonal of scenario space by simulation duration in days. Legend as in Figure 4b of the main text.

S2.15 Figure S15: Empirical data on transmission intensity during school closures

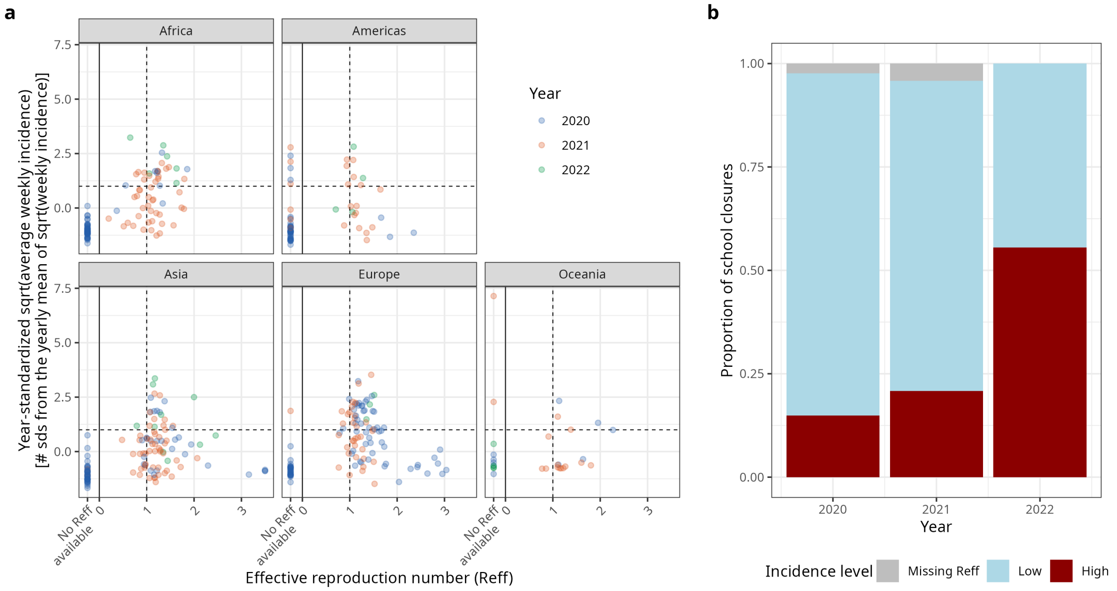

Figure S15: Analysis of school closure timings with respect to COVID-19 case incidence and effective reproduction number. a) Each point represents a school closure, and its position indicates the value of the effective reproduction number on the date of school closure (x axis), and the relative level of community incidence in that year (y axis). The relative incidence level is shown in terms of the year-standardized square root of the average weekly incidence on the date of school closures, where the value of 1 indicates 1 standard deviation away from the yearly mean weekly incidence. The upper-right quadrant defined by the dotted lines defines school closures that occurred in COVID-19 incidence periods: more than 1sd from the mean average weekly incidence and Reff ¿ 1. b) Proportion of school closures that occurred by community incidence level.

S2.16 Figure S16: Human mobility data for the state of Geneva

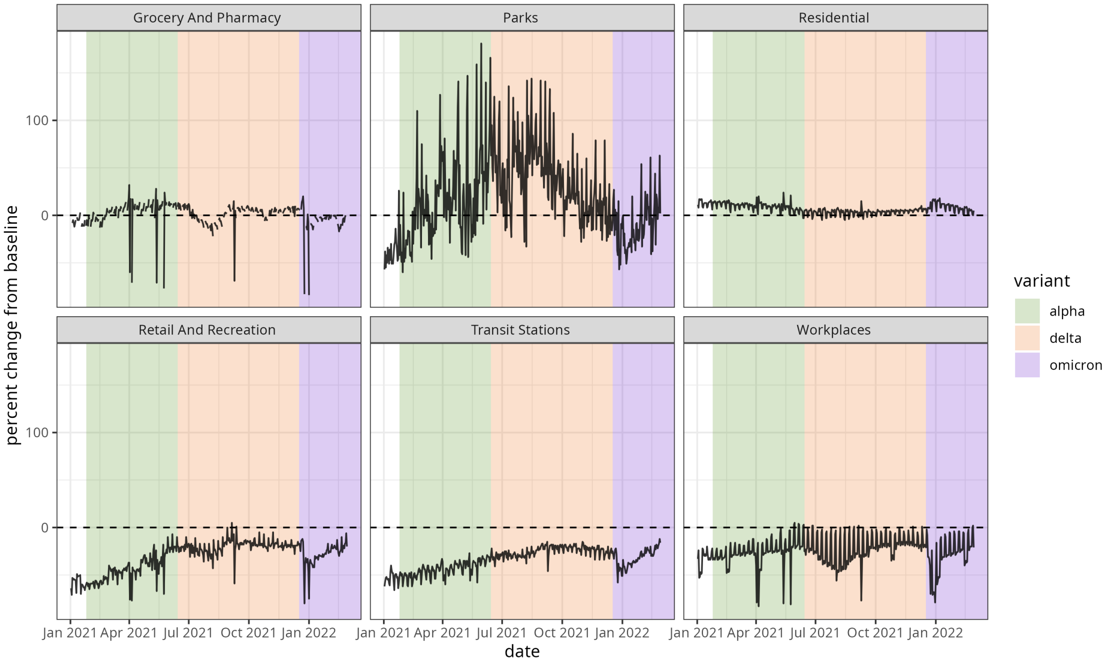

Figure S16: Human mobility data for the state of Geneva during the study period. Human mobility data consists of percent changes in activity with respect to baseline conditionsduring a five week period Jan 3–Feb 6, 2020 [35].

S3 Supplementary Tables

S3.1 Table S1: Baseline children

	School year 2020-2021				School year 2021-2022				
**School or nursery**	**School A**	**Pre-school C**	**Pre-school D**	**Total**	**School A**	**School B**	**Pre-school C**	**Pre-school D**	**Total**
n participant/N total (%)	124/156 (81%)	28/55 (51%)	50/64 (78%)	202/275 (73%)	149/185 (81%)	67/72 (93%)	21/37 (57%)	31/49 (63%)	268/343 (78%)
**Sex: n/n participant (%)**									
Female	62/124 (50%)	12/28 (43%)	23/50 (46%)	97/202 (48%)	75/149 (50%)	36/67 (54%)	8/21 (38%)	13/31 (42%)	132/268 (49%)
Male	62/124 (50%)	16/28 (57%)	27/50 (54%)	105/202 (52%)	74/149 (50%)	31/67 (46%)	13/21 (62%)	18/31 (58%)	136/268 (51%)
**Age[1] (years):**									
Median (IQR) [min-max]	5 (4-5) [3-7]	2.5 (2-3) [1-4]	3 (2-3) [1-4]	4 (3-5) ([1-7]	4 (3-5) [3-7]	5 (4-5) [3-6]	3 (2-3) [2-4]	3 (2-3) [2-4]	4 (3-5) [2-7]
**Physical health[2]: n/n completed questionnaire (%)**									
Very good	81/97 (84%)	20/24 (84%)	34/44 (77%)	135/165 (82%)	93/111 (84%)	37/51 (73%)	16/19 (84%)	22/29 (76%)	168/210 (80%)
Good	15/97 (15%)	2/24 (8%)	10/44 (23%)	27/165 (16%)	17/111 (15%)	14/51 (27%)	2/19 (11%)	7/29 (24%)	40/210 (19%)
Average	1/97 (1%)	2/24 (8%)	0	3/165 (2%)	1/111 (1%)	0	1/19 (5%)	0	2/210 (1%)
Bad / Very bad	0	0	0	0	0	0	0	0	0
**Chronic disease[3]: n/n completed questionnaire (%)**									
Yes	7/96 (7%)	2/24 (8%)	2/44 (5%)	11/164 (7%)	10/110 (9%)	2/51 (4%)	1/19 (5%)	1/29 (3%)	14/209 (7%)
**Baseline serology[4]: n/n tested (%)**									
Positive	19/90 (21%)	2/28 (7%)	13/47 (28%)	34/165 (21%)	3/31 (10%)	5/49 (10%)	-	-	8/80 (10%)
Undetermined	1/90 (1%)	1/28 (4%)	5/47 (10%)	7/165 (4%)	2/31 (6%)	4/49 (8%)	-	-	6/80 (8%)
Negative	70/90 (78%)	25/28 (89%)	29/47 (62%)	124/165 (75%)	26/31 (84%)	40/49 (82%)	-	-	66/80 (83%)
**SARS-CoV-2 infection diagnosed during the first year of the study[5]: n/n participant (%)**									
Yes	-	-	-	-	33/149 (22%)	-	0/21 (0%)	0/31 (0%)	33/201 (16%)

Figure S17: Table S1: -: not applicable, IQR: interquartile range. The total number of pupils in classes or groups was indicated with the variable 'N total'. An individual was considered a participant if they signed an informed consent form ('n participant').

[1]Ages were calculated on the day of the school's 1st baseline visit in the corresponding school year. (Year 1: March 2021 for School A, Pre-school C, and Pre-school D; Year 2: September 2021 for School A and Pre-school D, October 2021 for School B). For schools that didn't have a baseline visit in the 2nd school year, the ages were calculated on September 1st, 2021.
[2]Parent-reported.
[3]Parent-reported. Comorbidities included: kidney disease, alpha-1 antitrypsin deficiency, adenoid hypertrophy, eczema, pulmonary disease, hearing disorder, attention deficit hyperactivity disorder, cardiac disease, atopic dermatitis, cervical cystic lymphangioma, neuro-development disorder, sight disorder, celiac disease, asthma, unknown disease.
[4]Baseline serology: In the school year 2021-2022, serology was performed only at School B (new school) and at School A in the classes that joined the study that year.
[5]A previous SARS-CoV-2 infection was defined by a positive RT-PCR/RDT or a positive serology in a non-vaccinated participant. None of the children had been vaccinated against SARS-CoV-2 at the time of the baseline visits. In Geneva, vaccination was available from age 12 in July 2021 and from age 5 in January 2022.

S3.2 Table S2: Baseline adults

	School year 2020-2021				School year 2021-2022				
**School or nursery**	**School A**	**Pre-school C**	**Pre-school D**	**Total**	**School A**	**School B**	**Pre-school C**	**Pre-school D**	**Total**
n participant/N total (%)	11/12 (92%)	9/17 (53%)	10/14 (71%)	30/43 (70%)	11/11 (100%)	12/12 (100%)	10/11 (91%)	10/11 (91%)	43/45 (96%)
**Sex: n/n participant (%)**									
Female	11/11 (100%)	8/9 (89%)	8/10 (80%)	27/30 (90%)	11/11 (100%)	11/12 (92%)	9/10 (90%)	8/10 (80%)	39/43 (91%)
Male	0/11 /0%)	1/9 (11%)	2/10 (20%)	3/30 (10%)	0/11 (0%)	1/12 (8%)	1/10 (10%)	2/10 (20%)	4/43 (9%)
**Age[1] (years):**									
Median (IQR) [min-max]	42 (30-54) [26-58]	27 (26-31) [20-32]	35 (27-50) [23-56]	31 (27-47) [20-58]	43 (30-55) [27-59]	48 (35-52) [32-62]	30 (26-32) [20-33]	35 (27-51) [24-57]	35 (28-51) [20-62]
**Chronic disease[2]: n/n completed questionnaire (%)**									
Yes	4/10 (40%)	2/6 (33%)	1/9 (11%)	7/25 (28%)	4/10 (40%)	0/9 (0%)	2/6 (33%)	1/9 (11%)	7/34 (21%)
**Baseline serology[3]: n/n tested (%)**									
Positive	1/9 (11%)	3/8 (37%)	4/10 (40%)	8/27 (30%)	NA	10/12 (84%)	-	-	10/12 (84%)
Undetermined	0/9 (0%)	0/8 (0%)	0/10 (0%)	0/27 (0%)	NA	1/12 (8%)	-	-	1/12 (8%)
Negative	8/9 (89%)	5/8 (63%)	6/10 (60%)	19/27 (70%)	NA	1/12 (8%)	-	-	1/12 (8%)
**SARS-CoV-2 vaccination[4]: n/n eligible to vaccination (%)**									
At least one dose of vaccine	1/1 (100%)[5]	0/0	0/0	1/1 (100%)	2/11 (18%)	0/12 (0%)	1/10 (10%)	1/10 (10%)	4/43 (9%)
**SARS-CoV-2 infection diagnosed during the first year of the study[6]: n/n participant (%)**									
Yes	-	-	-	-	2/11 (27%)	-	0/10 (0%)	0/10 (0%)	2/31 (10%)

Figure S18: Table S2: -:not applicable, IQR: interquartile range. The total number of teachers and teaching assistants in the classes or groups included was indicated with the variable "N total". An individual was considered a participant if they signed an informed consent form ("n participant").

[1]Ages were calculated on the day of the school's 1st baseline visit in the corresponding school year. (Year 1: March 2021 for School A, Pre-school C, and Pre-school D; Year 2: September 2021 for School A and Pre-school D, October 2021 for School B). For schools that didn't have a baseline visit in the 2nd school year, the ages were calculated on September 1st, 2021.
[2]Self-reported. Comorbidities included: allergic, metabolic and psychiatric diseases, diabetes, obesity, digestive diseases, respiratory diseases, joint diseases, metabolic diseases.
[3]Baseline serology: In the school year 2021-2022, serology was only performed at School B (new school) and at School A in the classes that joined the study that year.
[4]We reported all participants who had at least 1 dose of SARS-CoV-2 vaccine before their school's baseline visit (or before September 1, 2021 for schools that didn't have a baseline visit on the 2nd school year). A participant was considered eligible for vaccination if they were in the age category that had access to the vaccine at that time in Geneva (https://www.ge.ch/document/covid-19-chiffres-campagne-vaccination-geneve). No criteria other than age were considered.
[5]This participant was vaccinated before vaccination was available for their age group because of a health condition.
[6]A previous SARS-CoV-2 infection was defined by a positive RT-PCR/RDT or a positive serology in a non-vaccinated participant.

S3.3 Table S3: Alpha outbreaks

Outbreak	Months	Variant	School	Index case	Class	Participants	Serology at the beginning of the outbreak (D0)	Vaccinal status at the beginning of the outbreak	Positive RT-PCR	Seroconversion without positive RT-PCR	Total SARS-CoV-2 cases	Symptoms
						(age min-max) n investigated/n participant/N total	n positive serology/n tested (n undetermined serology/n tested)	n vaccinated/ n eligible for vaccination[1]	n positive RT-PCR or RDT/n tested[2]	n seroconversion between D0 and D30 without positive RT-PCR/n with a serology at D0 and at D30	n confirmed cases/n investigated	n symptomatic/n confirmed
#1[3]	March 2021	Unknown	SC	approx. 24/03/2021	Class 7	Children (1-3 y.o.): 18/18/20	1/18 (1/18)	0/0	0/8	0/5	0/18	-
						Teachers (20-31 y.o.): 7/7/7	3/7 (0/7)	0/0	0/2	0/2	0/7	-
						Total: 25/25/27	4/25 (1/25)	0/0	0/10	0/7	0/25	-
					Household	Adults: 0/0/0	-	-	-	-	-	-
						Children: 0/0/0	-	-	-	-	-	-
						Total in households: 0/0/0	-	-	-	-	-	-
#2	April 2021	Alpha	SA[4]	26/04/2021	Class 9	Children (2-4 y.o.): 21/21/21	4/18 (0/18)	0/0	9/20	4/17	13/21	7/13
						Adults (27-58 y.o.): 2/2/2	0/2 (0/2)	1/1	0/2	0/2	0/2	-
						Total: 23/23/23	4/20 (0/20)	1/1	9/22	4/19	13/23	7/13
					Class 10	Children (2-4 y.o.): 17/19/21[5]	2/14 (0/14)	0/0	1/17	0/11	1/17	1/1
						Adults (26-46 y.o.): 2/2/2	0/0	0/1	1/1	-	1/2	1/1
						Total: 19/21/23	2/14 (0/14)	0/1	2/18	0/11	2/19	2/2
				04/05/2021	Class 15	Children (4-6 y.o.): 18/18/18	4/15 (2/15)	0/0	1/18	0/14[6]	1/18	0/1
						Adults (56 y.o.): 1/1/1	0/1 (0/1)	0/1	0/1	1/1	1/1	0/1
						Total: 19/19/19	4/16 (2/16)	0/1	1/19	1/15	2/19	0/2
					Class 16	Children (4-6 y.o.): 16/16/17	1/12 (1/12)	0/0	0/16	1/9[7]	1/16	1/1
						Adults (54 y.o.): 1/1/1	0/1 (0/1)	0/1	0/1	0/1	0/1	-
						Total: 17/17/18	1/13 (1/13)	0/1	0/17	1/10	1/17	1/1
					All investigated classes	Children (2-6 y.o.): 72/74/77	11/59 (3/59)	0/0	11/63	5/51	16/72	9/14
						Adults (26-58 y.o.): 6/6/6	0/4 (0/4)	1/4	1/5	1/4	2/6	1/2
						Total: 78/80/83	11/63 (3/63)	1/4	12/76	6/55	18/78	10/16
					Households	Children (1-8 y.o.): 4/4/4	0/3 (0/3)	0/0	0/4	0/3	0/4	-
						Adults (20-49 y.o.): 17/17/18	3/15 (3/15)	0/2	1/17	0/15[8]	1/17	0/1
						Total: 21/21/22	3/18 (3/18)	0/2	1/21	0/18	1/21	0/1

Figure S19: Table S3: -: not applicable, y.o.: years old, RT-PCR: reverse transcription polymerase chain reaction, RDT: rapid diagnostic test. The total number of pupils and teachers in the classes or groups included was indicated with the variable 'N total'. In households, 'N total' represents the number of people living in the household of a SARS-CoV-2 positive child. An individual was considered a participant if they had signed an informed consent form ('n participant'). A participant was considered to have been investigated in an outbreak ('n investigated') if they had had at least one RT-PCR or RDT within 21 days of identification of the index case and/or at least one serology during the outbreak visits. The number of participants tested by a given test (serology at D0 or RT-PCR/RDT) is represented by the variable 'n tested'. Only data from participants (with a signed consent form) were presented in this table. Participants under 18 years of age were considered children. The age of the participants was calculated on the day of the 1st visit of the outbreak in their class/household.

[1]Eligible to vaccination according to the vaccination dates in Geneva (https://www.ge.ch/document/covid-19-chiffres-campagne-vaccination-geneve) and the age of the participants.
[2]Only tests done within the 21 days after the index case identification of the class or after the child index case identification of the household.
[3]Index case didn't participate to the study and is not reported in this table. Viral sequencing could not be performed in this outbreak. During this period the Alpha variant was largely dominant in Geneva region.
[4]In this school, the teachers work in pairs, alternating every other week in 2 parallel classes. One member of each pair has been assigned to one of the 2 classes in this table.
[5] Three of the 28 participants were not exposed to this outbreak because they did not attend school during that period. They were excluded from the investigation.
[6] Two participants possibly seroconverted because of an infection but were not counted because they did not meet the criteria of our definition of seroconversion. The first one had an undetermined serology at D0 and a positive one at D30. The second one did not have a serology at D0 but had a positive one at D30.
[7] A participant had an undetermined serology at D0 and a positive one at D30.
[8]A household member had a undetermined serology at D0 and a positive one at D30, without vaccination in between.

S3.4 Table S4: Delta outbreaks

Outbreak	Months	Variant	School	Index case	Class	Participants	Serology at the beginning of the outbreak (D0)	Vaccinal status at the beginning of the outbreak	Positive RT-PCR	Seroconversion without positive RT-PCR	Total SARS-CoV-2 cases	Symptoms
						(age min-max) n investigated/n participant/N total	n positive serology/n tested (n undetermined serology/n tested)	n vaccinated/ n eligible for vaccination[1]	n positive RT-PCR or RDT/n tested[2]	n seroconversion between D0 and D30 without positive RT-PCR/n with a serology at D0 and at D30	n confirmed cases/n investigated	n symptomatic/n confirmed
#3[3]	July 2021	Delta	SD	approx. 14/07/2021	Class 2	Children (2 y.o.): 8/12/18[4]	1/1 (0/1)	0/0	2/7	0/0	2/8	2/2
						Teachers (42-50 y.o.): 2/2/2	0/0 (0/0)	0/2	1/2	-	1/2	NA
						Total: 9/13/19	1/1 (0/1)	0/1	2/13	0/0	2/9	2/2
					Household	Adults (33 y.o.): 1/4/4	0/0 (0/0)	4/4	1/1	-	1/1	NA
						Children (3 y.o.): 1/2/2	0/0 (0/0)	0/0	0/1	-	0/1	-
						Total in households: 2/6/6	0/0 (0/0)	4/4	1/2	-	1/2	NA
#4	October 2021	Delta	SA	01/10/2021	Class 9	Children (3-4 y.o.): 14/14/19	1/11 (1/11)	0/0	7/14	0/10	7/14	4/7
						Adults (43-59 y.o.): 2/2/2	0/0 (0/0)	1/2	1/2	-	1/2	1/1
						Total: 16/16/21	1/11 (1/11)	1/2	8/16	0/10	8/16	5/8
					Class 10	Children (3-5 y.o.): 14/14/19[5]	0/9 (0/9)	0/0	1/14	2/7	3/14	0/3
						Adults (27-48 y.o.): 2/2/2	0/1 (1/1)	0/2	0/2	0/0	0/2	-
						Total: 16/16/21	0/10 (1/10)	0/2	1/16	2/7	3/16	0/3
				04/10/2021	Class 11	Children (3-4 y.o.): 18/18/21	3/15 (3/15)	0/0	0/17	0/13	0/18	-
						Adults (39 y.o.): 1/1/1	0/0 (0/0)	0/1	1/1	0/0	1/1	1/1
						Total: 19/19/22	3/15 (3/15)	0/1	1/18	0/13	1/19	1/1
					Class 14	Children (4-6 y.o.): 9/12/16	1/5 (2/5)	0/0	1/9	0/4	1/9	0/1
						Adults (28 y.o.): 1/1/1	0/0 (0/0)	1/1	0/1	-	0/1	-
						Total: 10/13/17	1/5 (2/5)	1/1	1/10	0/4	1/10	0/1
					All investigated classes	Children (3-6 y.o.): 55/58/75	5/40 (6/40)	0/0	9/54	2/34	11/55	4/11
						Adults (27-59 y.o.): 6/6/6	0/1 (1/1)	2/6	2/6	0/0	2/6	2/2
						Total: 61/64/81	5/41 (7/41)	2/6	11/60	2/34	13/61	6/13
					Households	Children (0-7 y.o.): 7/7/7	1/6 (2/6)	0/0	2/7	0/5	2/7	2/2
						Adults (34-45 y.o.): 16/16/16	13/15 (1/15)	12/16	2/16	0/12	2/16	2/2
						Total: 23/23/23	14/21 (3/21)	12/16	4/23	0/17	4/23	4/4
#5	November 2021	Delta	SA	26/11/2021	Class 15	Children (5-7 y.o.): 17/20/21	3/13 (1/13)	0/0	1/17	0/4	1/17	1/1
						Adults (57 y.o.): 0/1/1	-	0/1	-	-	-	-
						Total: 17/19/22	3/13 (1/13)	0/1	1/17	0/4	1/17	1/1
					Class 16	Children (5-7 y.o.): 18/19/21	1/16 (0/16)	0/0	4/16	0/1	4/18	3/4
						Adults (55 y.o.): 0/1/1	-	0/1	-	-	-	-
						Total: 18/20/22	1/16 (0/16)	0/1	4/16	0/1	4/18	3/4
					All investigated classes	Children (5-7 y.o.): 35/49/42	4/29 (1/29)	0/0	5/33	0/5	5/35	4/5
						Adults (55-57 y.o.): 0/2/2	-	0/2	-	-	-	-
						Total: 35/51/44	4/29 (1/29)	0/2	5/33	0/5	5/35	4/5
					Households	Children (0-8 y.o.): 4/4/4	0/1 (0/1)	0/0	2/4	0/1	2/4	2/2
						Adults (32-44 y.o.): 9/9/12	7/7 (0/7)	5/9	4/9	0/0	4/9	4/4
						Total: 13/13/16	7/8 (0/8)	5/9	6/13	0/1	6/13	6/6
#6	December 2021	Delta	SD	04/12/2021	Class 5	Children (3-4 y.o.): 18/20/25	4/14 (0/14)	0/0	3/18	0/6	3/18	1/3
						Teachers (26-48 y.o.): 3/4/5	0/0 (0/0)	0/4	1/3	-	1/3	1/1
						Total: 21/24/30	4/14 (0/14)	0/4	4/21	0/6	4/21	2/4
					Household	Adults (37-71 y.o.): 7/7/7	5/5 (0/5)	3/7	0/7	0/4	0/7	-
						Children (0-6 y.o.): 2/2/2	0/1 (0/1)	0/0	0/2	0/1	0/2	-
						Total in households: 9/9/9	5/6 (0/6)	3/7	0/9	0/5	0/9	-
#7	December 2021	Delta	SB	07/12/2021	Class 19	Children (3-4 y.o.): 15/16/18	0/10 (1/10)	0/0	2/12	0/6	2/15	1/2
						Adults (35-54 y.o.): 4/4/4	4/4 (0/4)	0/4	0/4	0/0	0/4	-
						Total: 19/20/22	4/14 (1/14)	0/4	2/16	0/6	2/19	1/2
					Class 20	Children (4-5 y.o.): 16/17/18	0/12 (0/12)	0/0	3/15	0/6	3/16	3/3
						Adults (38-61 y.o.): 2/2/2	1/1 (0/1)	0/2	0/2	0/0	0/2	-
						Total: 18/19/20	1/13 (0/13)	0/2	3/17	0/6	3/18	3/3
					Class 21	Children (4-6 y.o.): 16/16/18	4/14 (1/14)	0/0	1/15	1/4	2/16	0/2
						Adults (32-62 y.o.): 4/4/4	2/4 (0/4)	0/4	0/4	0/2	0/4	-
						Total: 20/20/22	6/18 (1/18)	0/4	1/19	1/6	2/20	0/2
					Class 22	Children (5-7 y.o.): 18/18/18	2/15 (0/15)	0/0	3/18	0/13	3/18	2/3
						Adults (45-50 y.o.): 2/2/2	2/2 (0/2)	0/2	1/2	0/0	1/2	1/1
						Total: 20/20/20	4/17 (0/17)	0/2	4/20	0/13	4/20	3/4
					All investigated classes	Children (3-7 y.o.): 65/67/72	6/51 (2/51)	0/0	9/60	1/29	10/66	6/10
						Adults (32-62 y.o.): 12/12/12	9/11 (0/11)	0/12	1/12	0/2	1/12	1/1
						Total: 77/79/84	15/62 (2/62)	0/12	10/72	1/31	11/78	7/11
					Households	Children (5-12 y.o.): 5/7/7	3/3 (0/3)	0/1	3/5	0/0	3/5	2/3
						Adults (37-52 y.o.): 14/15/16	3/4 (0/4)	7/15	3/14	0/1	3/14	2/3
						Total: 19/22/23[6]	6/7 (0/7)	7/16	6/19	0/1	6/19	4/6
#8	December 2021	Delta	SA	10/12/2021	Class 14	Children (4-5 y.o.): 6/13/16	0/0 (0/0)	0/0	0/5	-	0/6	-
						Adults (28-39 y.o.): 0/2/2	0/0 (0/0)	1/2	0/0	-	-	-
						Total: 6/15/18	0/0 (0/0)	1/2	0/5	-	0/6	-
					Households	Adults: 0/0/0	-	-	-	-	-	-
						Children: 0/0/0	-	-	-	-	-	-
						Total: 0/0/0	-	-	-	-	-	-

Figure S20: Table S4: -: not applicable, NA: information not available, y.o.: years old, RT-PCR: reverse transcription polymerase chain reaction, RDT: rapid diagnostic test. The total number of pupils and teachers in the classes or groups included was indicated with the variable "N total". In households, "N total" represents the number of people living in the household of a SARS-CoV-2 positive child. An individual was considered a participant if they had signed an informed consent form ("n participant"). A participant was considered to have been investigated in an outbreak ("n investigated") if they had had at least one RT-PCR or RDT within 21 days of identification of the index case and/or at least one serology during the outbreak visits. The number of participants tested by a given test (serology at D0 or RT-PCR/RDT) is represented by the variable 'n tested'. Only data from participants (with a signed consent form) were presented in this table. Participants under 18 years of age were considered children. The age of the participants was calculated on the day of the 1st visit of the outbreak in their class/household.

[1]Eligible to vaccination according to the vaccination dates in Geneva (https://www.ge.ch/document/covid-19-chiffres-campagne-vaccination-geneve) and the age of the participants.
[2]Only tests done within the 21 days after the index case identification of the class or after the child index case identification of the household.
[3] Two other teachers of other groups in this pre-school were tested positive by RT-PCR during this outbreak.
[4]Three of the 12 participants were not exposed to this outbreak because they did not attend preschool during that period. They were excluded from the investigation.
[5] One of the 26 participants were not exposed to this outbreak because they did not attend school during that period. They were excluded from the investigation.
[6] Three household members (2 adults and 1 child) were tested positive before the participating child (respectively 7 days, 1 day and 7 days before). They were excluded from the investigation.

S3.5 Table S5: Omicron BA.1 outbreaks

Outbreak	Months	Variant	School	Index case	Class	Participants	Serology at the beginning of the outbreak (D0)	Vaccinal status at the beginning of the outbreak	Positive RT-PCR	Seroconversion without positive RT-PCR	Total SARS-CoV-2 cases	Symptoms
						(age min-max) n investigated/n participant/N total	n positive serology/n tested (n undetermined serology/n tested)	n vaccinated/ n eligible for vaccination[1]	n positive RT-PCR or RDT/n tested[2]	n seroconversion between D0 and D30 without positive RT-PCR/n with a serology at D0 and at D30	n confirmed cases/n investigated	n symptomatic/n confirmed
#9	January 2022	Omicron BA.1	SB	11/01/2022	Class 19	Children (3-4 y.o.): 12/16/18	4/9 (0/9)	0/0	4/12	1/7	5/12	4/5
						Adults (35-54 y.o.): 3/4/4	0/0 (0/0)	0/4	1/3	-	1/3	1/1
						Total: 15/20/22	4/9 (0/9)	0/4	5/15	1/7	6/15	5/6
					Class 20	Children (4-5 y.o.): 13/17/18	1/8 (0/8)	0/0	1/12	0/6	1/13	1/1
						Adults (38-62 y.o.): 2/2/2	0/0 (0/0)	0/2	1/2	-	1/2	1/1
						Total: 15/19/20	1/8 (0/8)	0/2	2/14	0/6	2/15	2/2
					Class 21	Children (5-6 y.o.): 15/16/18	2/5 (0/5)	0/13	9/15	1/3	10/15	5/10
						Adults (32-62 y.o.): 4/4/4	0/2 (0/2)	0/4	2/4	0/0	2/4	2/2
						Total: 19/20/22	2/7 (0/7)	0/17	11/19	1/3	12/19	7/12
					Class 22	Children (5-7 y.o.): 18/18/18	3/14 (0/14)	0/17	11/18	1/7	12/18	6/12
						Adults (46-50 y.o.): 2/2/2	0/0 (0/0)	0/2	1/2	-	1/2	1/1
						Total: 20/20/20	3/14 (0/14)	0/19	12/20	1/7	13/20	7/13
					All investigated classes	Children (3-7 y.o.): 58/67/72	10/36 (0/36)	0/30	25/57	3/23	28/58	16/28
						Adults (32-62 y.o.): 11/12/12	0/2 (0/2)	0/12	5/11	0/0	5/11	5/5
						Total: 69/79/84	10/38 (0/38)	0/42	30/68	3/23	33/69	21/33
					Households	Children (1-11 y.o.): 14/15/16	2/6 (0/6)	0/0	7/13	0/0	7/14	2/7
						Adults (19-57 y.o.) : 40/46/50	5/5 (0/5)	23/46	16/40	0/1	16/40	10/16
						Total: 54/61/66[3]	7/11 (0/11)	23/46	23/53	0/2	23/54	12/23
#10	January 2022	Omicron BA.1	SC	09/01/2022	Class 8	Children (3-5 y.o.): 10/19/19	0/7 (1/7)	0/0	4/9	0/3	4/10	3/4
						Teachers (25-31 y.o.): 1/3/4	0/0 (0/0)	0/3	1/1	-	1/1	NA
						Total: 11/22/23	0/7 (1/7)	0/3	5/10	0/3	5/11	3/4
					Household	Adults (33-37 y.o.): 5/6/6[4]	0/0 (0/0)	2/6	2/5	-	2/5	2/2
						Children (0-1 y.o.): 2/2/2	0/0 (0/0)	0/0	0/1	-	0/2	-
						Total in households: 7/8/6	0/0 (0/0)	2/6	2/6	-	2/7	2/2
#11	January 2022	Omicron BA.1	SA	14/01/2022	Class 16	Children (5-6 y.o.): 11/20/21	2/5 (0/5)	0/15	4/11	0/0	4/11	4/4
						Adults (55-57 y.o.): 1/2/2	0/0 (0/0)	0/2	0/1	-	0/1	-
						Total: 12/22/23	2/5 (0/5)	0/17	4/12	0/0	4/12	4/4
				08/01/2022	Class 17	Children (6-8 y.o.): 9/13/17	1/4 (0/4)	0/21	1/6	0/0	1/9	0/1
						Adults (39 y.o.): 0/1/1	-	0/1	-	-	-	-
						Total: 9/14/18	1/4 (0/4)	0/22	1/6	0/0	1/9	0/1
				13/01/2022	Class 18	Children (6-8 y.o.): 9/16/18	3/5 (0/5)	0/23	5/7	0/2	5/9	2/5
						Adults (31 y.o.): 1/1/1	0/0 (0/0)	0/1	1/1	-	1/1	0/1
						Total: 9/17/19	3/5 (0/5)	0/24	6/8	0/2	6/10	1/6
					All investigated classes	Children (5-8 y.o.): 28/49/56	6/14 (0/14)	0/59	9/23	0/2	9/28	6/9
						Adults (31-57 y.o.): 2/4/4	0/0 (0/0)	0/4	1/2	-	1/2	0/1
						Total: 30/53/60	6/14 (0/14)	0/63	10/25	0/2	10/30	6/10
					Households	Children (2-11 y.o.): 4/4/4	0/0 (0/0)	0/2	4/4	-	4/4	1/4
						Adults (30-52 y.o.): 9/10/17	0/0 (0/0)	5/10	1/9	-	1/9	1/1
						Total: 13/14/21	0/0 (0/0)	5/12	5/13	-	5/13	2/5

Figure S21: Table S5: -: not applicable, NA: information not available, y.o.: years old, RT-PCR: reverse transcription polymerase chain reaction, RDT: rapid diagnostic test. The total number of pupils and teachers in the classes or groups included was indicated with the variable "N total". In households, "N total" represents the number of people living in the household of a SARS-CoV-2 positive child. An individual was considered a participant if they had signed an informed consent form ("n participant"). A participant was considered to have been investigated in an outbreak ("n investigated") if they had had at least one RT-PCR or RDT within 21 days of identification of the index case and/or at least one serology during the outbreak visits. The number of participants tested by a given test (serology at D0 or RT-PCR/RDT) is represented by the variable 'n tested'. Only data from participants (with a signed consent form) were presented in this table. Participants under 18 years of age were considered children. The age of the participants was calculated on the day of the 1st visit of the outbreak in their class/household.

[1] Eligible to vaccination according to the vaccination dates in Geneva (https://www.ge.ch/document/covid-19-chiffres-campagne-vaccination-geneve) and the age of the participants.
[2] Only tests done within the 21 days after the index case identification of the class or after the child index case identification of the household.
[3] Five household members (5 adults) were tested positive before the participating child (respectively 1 day, 4 days, 6 days, 1 day and 4 days before). They were excluded from the investigation.
[4] A parent tested positive one day before his/her child. The positive test is not reported in the table and this parent is considered 'not investigated'.

S3.6 Table S6: GISAID sequences - Alpha

SUPPLEMENTAL TABLE

Data Availability

GISAID Identifier:	EPI_SET_240117cm
doi:	10.55876/gis8.240117cm

All genome sequences and associated metadata in this dataset are published in GISAID's EpiCoV database. To view the contributors of each individual sequence with details such as accession number, Virus name, Collection date, Originating Lab and Submitting Lab and the list of Authors, visit 10.55876/gis8.240117cm

Data Snapshot

- EPI_SET_240117cm is composed of 5,156 individual genome sequences.
- The collection dates range from 2019-12-24 to 2021-06-10;
- Data were collected in 85 countries and territories;
- All sequences in this dataset are compared relative to hCoV-19/Wuhan/WIV04/2019 (WIV04), the official reference sequence employed by GISAID (EPI_ISL_402124). Learn more at https://gisaid.org/WIV04.

S3.7 Table S7: GISAID sequences - Delta/Omicron BA.1

SUPPLEMENTAL TABLE

Data Availability

GISAID Identifier:	EPI_SET_240117ea
doi:	10.55876/gis8.240117ea

All genome sequences and associated metadata in this dataset are published in GISAID's EpiCoV database. To view the contributors of each individual sequence with details such as accession number, Virus name, Collection date, Originating Lab and Submitting Lab and the list of Authors, visit 10.55876/gis8.240117ea

Data Snapshot

- EPI_SET_240117ea is composed of 5,156 individual genome sequences.
- The collection dates range from 2019-12-24 to 2021-06-10;
- Data were collected in 85 countries and territories;
- All sequences in this dataset are compared relative to hCoV-19/Wuhan/WIV04/2019 (WIV04), the official reference sequence employed by GISAID (EPI_ISL_402124). Learn more at https://gisaid.org/WIV04.

S3.8 Table S8: Parameter values of dynamic model

parameter	description	value by variant			units	reference
		alpha	delta	omicron		
ϕ	transition rate from exposed and pre-symptomatic compartments	1.10	0.97	0.77	day^{-1}	computed from [13] and [36]
k_E	number of exposed compartments	1	1	1	-	computed from [13] and [36]
k_P	number of pre-symptomatic compartments	3	3	3	-	computed from [13] and [36]
γ	transition rate from symptomatic compartment	0.28	0.29	0.29	day^{-1}	computed from [13] and [36]
k_I	number of symptomatic compartments	1	1	1	-	computed from [13] and [36]
α	relative infectivity of pre-symptomatic vs. symptomatic	2.80	3.90	3.90	-	[13]
β_C	hazard of infection from community	0.044 (0.038-0.054)	0.28 (0.17-0.39)	9.5 (7- 12)	$year^{-1}$	estimated
β_g	hazard of infection from same class	1.1 (0.2-1.4)	1 (0.77-1.3)	1.3 (0.94-1.6)	$infector^{-1}$ $year^{-1}$	estimated
β_s	hazard of infection between classes in same school	0.092 (0.036-0.15)	0.057 (0.017-0.096)	0.4 (0.3-0.48)	$infector^{-1}$ $year^{-1}$	estimated

Table S8: Parameter values used in dynamic model

S3.9 Table S9: Genbank accession numbers of sequences generated in this study

GISAID name	Genbank name	Genbank accession number
hCoV-19/Switzerland/GE-CEVD-19102122/2021	Switzerland/GE-CEVD-19102122/2021	PV687587
hCoV-19/Switzerland/GE-CEVD-63059400/2021	Switzerland/GE-CEVD-63059400/2021	PV687576
hCoV-19/Switzerland/GE-CEVD-65092500/2022	Switzerland/GE-CEVD-65092500/2022	PV687590
hCoV-19/Switzerland/GE-CEVD-65092504/2022	Switzerland/GE-CEVD-65092504/2022	PV687608
hCoV-19/Switzerland/GE-CEVD-72015600/2021	Switzerland/GE-CEVD-72015600/2021	PV687615
hCoV-19/Switzerland/GE-CEVD-72015800/2021	Switzerland/GE-CEVD-72015800/2021	PV687614
hCoV-19/Switzerland/GE-CEVD-72016400/2021	Switzerland/GE-CEVD-72016400/2021	PV687613
hCoV-19/Switzerland/GE-CEVD-72016800/2021	Switzerland/GE-CEVD-72016800/2021	PV687612
hCoV-19/Switzerland/GE-CEVD-72021200/2022	Switzerland/GE-CEVD-72022100/2021	PV687577
hCoV-19/Switzerland/GE-CEVD-72025710/2021	Switzerland/GE-CEVD-72025710/2021	PV687581
hCoV-19/Switzerland/GE-CEVD-72026200/2022	Switzerland/GE-CEVD-72026200/2022	PV687606
hCoV-19/Switzerland/GE-CEVD-72027200/2021	Switzerland/GE-CEVD-72027200/2021	PV687580
hCoV-19/Switzerland/GE-CEVD-72027211/2021	Switzerland/GE-CEVD-72027211/2021	PV687579
hCoV-19/Switzerland/GE-CEVD-72029002/2021	Switzerland/GE-CEVD-72029002/2021	PV687586
hCoV-19/Switzerland/GE-CEVD-72029120/2021	Switzerland/GE-CEVD-72029120/2021	PV687575
hCoV-19/Switzerland/GE-CEVD-72029420j/2022	Switzerland/GE-CEVD-72029420j/2022	PV687605
hCoV-19/Switzerland/GE-CEVD-72030520/2021	Switzerland/GE-CEVD-72030520/2021	PV687578
hCoV-19/Switzerland/GE-CEVD-72059000/2022	Switzerland/GE-CEVD-72059000/2022	PV687609
hCoV-19/Switzerland/GE-CEVD-72059001/2022	Switzerland/GE-CEVD-72059001/2022	PV687596
hCoV-19/Switzerland/GE-CEVD-72059010/2022	Switzerland/GE-CEVD-72059010/2022	PV687610
hCoV-19/Switzerland/GE-CEVD-72060800/2021	Switzerland/GE-CEVD-72060800/2021	PV687582
hCoV-19/Switzerland/GE-CEVD-72060810/2021	Switzerland/GE-CEVD-72060810/2021	PV687585
hCoV-19/Switzerland/GE-CEVD-72061400b/2021	Switzerland/GE-CEVD-72061400b/2021	PV687589
hCoV-19/Switzerland/GE-CEVD-72061500/2022	Switzerland/GE-CEVD-72061500/2022	PV687597
hCoV-19/Switzerland/GE-CEVD-72061510/2022	Switzerland/GE-CEVD-72061510/2022	PV687598
hCoV-19/Switzerland/GE-CEVD-72061901/2021	Switzerland/GE-CEVD-72061901/2021	PV687583
hCoV-19/Switzerland/GE-CEVD-72062700/2022	Switzerland/GE-CEVD-72062700/2022	PV687591
hCoV-19/Switzerland/GE-CEVD-72063400/2022	Switzerland/GE-CEVD-72063400/2022	PV687592
hCoV-19/Switzerland/GE-CEVD-72063410/2022	Switzerland/GE-CEVD-72063410/2022	PV687599
hCoV-19/Switzerland/GE-CEVD-72063700/2021	Switzerland/GE-CEVD-72063700/2021	PV687584
hCoV-19/Switzerland/GE-CEVD-72064000/2022	Switzerland/GE-CEVD-72064000/2022	PV687600
hCoV-19/Switzerland/GE-CEVD-72064100/2022	Switzerland/GE-CEVD-72064100/2022	PV687593
hCoV-19/Switzerland/GE-CEVD-72064110/2022	Switzerland/GE-CEVD-72064110/2022	PV687601
hCoV-19/Switzerland/GE-CEVD-72064300/2022	Switzerland/GE-CEVD-72064300/2022	PV687611
hCoV-19/Switzerland/GE-CEVD-72064400/2022	Switzerland/GE-CEVD-72064400/2022	PV687602
hCoV-19/Switzerland/GE-CEVD-72064700/2021	Switzerland/GE-CEVD-72064700/2021	PV687588
hCoV-19/Switzerland/GE-CEVD-72064800/2022	Switzerland/GE-CEVD-72064800/2022	PV687603
hCoV-19/Switzerland/GE-CEVD-72065000/2022	Switzerland/GE-CEVD-72065000/2022	PV687594
hCoV-19/Switzerland/GE-CEVD-72065400/2022	Switzerland/GE-CEVD-72065400/2022	PV687595
hCoV-19/Switzerland/GE-CEVD-72065501/2022	Switzerland/GE-CEVD-72065501/2022	PV687604
hCoV-19/Switzerland/GE-CEVD-72067120/2022	Switzerland/GE-CEVD-72072300/2022	PV687607
hCoV-19/Switzerland/GE-HUG-34004381/2021	Switzerland/GE-HUG-34004381/2021	PV716594
hCoV-19/Switzerland/GE-HUG-34040922/2021	Switzerland/GE-HUG-34040922/2021	PV716598
hCoV-19/Switzerland/GE-HUG-34082801/2021	Switzerland/GE-HUG-34082801/2021	PV716599
hCoV-19/Switzerland/un-HUG-33984146/2021	Switzerland/un-HUG-33984146/2021	PV716596
hCoV-19/Switzerland/GE-HUG-34025441/2021	Switzerland/GE-HUG-34025441/2021	PV716595
hCoV-19/Switzerland/GE-HUG-34040929/2021	Switzerland/GE-HUG-34040929/2021	PV716597
hCoV-19/Switzerland/GE-HUG-34780782/2021	Switzerland/GE-HUG-34780782/2021	PV716600
hCoV-19/Switzerland/GE-HUG-34791602/2021	Switzerland/GE-HUG-34791602/2021	PV716601
hCoV-19/Switzerland/GE-HUG-35599505/2021	Switzerland/GE-HUG-35599505/2021	PV716603
hCoV-19/Switzerland/GE-HUG-35599579/2021	Switzerland/GE-HUG-35599579/2021	PV716602
hCoV-19/Switzerland/GE-HUG-35735460/2021	Switzerland/GE-HUG-35735460/2021	PV716604
hCoV-19/Switzerland/GE-HUG-36217799/2021	Switzerland/GE-HUG-36217799/2021	PV716605
hCoV-19/Switzerland/GE-HUG-36330789/2021	Switzerland/GE-HUG-36330789/2021	PV716606
hCoV-19/Switzerland/GE-HUG-36356700/2021	Switzerland/GE-HUG-36356700/2021	PV716607
hCoV-19/Switzerland/GE-HUG-36450688/2021	Switzerland/GE-HUG-36450688/2021	PV716608
hCoV-19/Switzerland/GE-HUG-36615497/2022	Switzerland/GE-HUG-36615497/2022	PV716609
hCoV-19/Switzerland/GE-HUG-36825943/2022	Switzerland/GE-HUG-36825943/2022	PV716610

S3.10 Table S10: GISAID DOI of sequences generated in this study

SUPPLEMENTAL TABLE

Data Availability

GISAID Identifier:	EPI_SET_250626bw
DOI:	https://doi.org/10.55876/gis8.250626bw

All genome sequences and associated metadata in this dataset are published in GISAID's EpiCoV database. To view the contributors of each individual sequence with details such as accession number, Virus name, Collection date, Originating Lab and Submitting Lab and the list of Authors, visit 10.55876/gis8.250107tk

Data Snapshot

EPI_SET_250626bw is composed of 45 individual genome sequences.
The collection dates range from 2021-04-27 to 2022-01-21;
Data were collected in 1 countries and territories.

S4 The SEROCoV-Schools study group

Elsa Lorthe, Julie Berthelot, Maria-Eugenia Zaballa, Hélène Baysson, Andrea Jutta Loizeau, Ania Wisniak, Stéphanie Testini, Khadija Samir, Natalie Francioli, Severine Harnal, Javier Perez-Saez, Nick Pullen, Francesco Pennacchio, Julien Lamour, Gaëlle Bryand-Rumley, Claire Semaani, Viviane Richard, Roxane Dumont, Prune Collombet, Natacha Noël, Patrick Bleich, Nacira El Merjani, Caroline Pugin, Jessica Rizzo, Marion Frangville, Antoine Bal, Fanny-Blanche Lombard, Zo Francia Randrianandrasana, Oumar Aly Ba, Chantal Martinez, Paola D'Ippolito, Camille Tible, Viola Bucolli, Livia Boehm, Adrien Jos Rastello, Lucie Ménard, Lison Beigbeder, Fréderic Rinaldi, Alain Cudet, Alexandre Moulin, Andrew S Azman, Arnaud G L'Huillier, Klara M Posfay-Barbe, Idris Guessous, Silvia Stringhini, Grégoire Michielin, Sebastian Maerkl, Fatemeh Arefi, Mathilde Bellon, Isabella Eckerle, Laurent Kaiser, Benjamin Meyer, Meriem Bekliz, Florian Laubscher, Gil Barbosa Monteiro

References

[1] Stefan Elbe and Gemma Buckland-Merrett. “Data, disease and diplomacy: GISAID’s innovative contribution to global health”. In: *Global challenges* 1.1 (2017), pp. 33–46.

[2] Emma B Hodcroft et al. “Spread of a SARS-CoV-2 variant through Europe in the summer of 2020”. In: *Nature* 595.7869 (2021), pp. 707–712.

[3] Silvia Stringhini et al. “Seroprevalence of anti-SARS-CoV-2 antibodies after the second pandemic peak”. In: *The Lancet Infectious Diseases* 21.5 (2021), pp. 600–601.

[4] Silvia Stringhini et al. “Seroprevalence of anti-SARS-CoV-2 antibodies 6 months into the vaccination campaign in Geneva, Switzerland, 1 June to 7 July 2021”. In: *Eurosurveillance* 26.43 (2021), p. 2100830.

[5] Maria-Eugenia Zaballa et al. “Seroprevalence of anti-SARS-CoV-2 antibodies and cross-variant neutralization capacity after the Omicron BA. 2 wave in Geneva, Switzerland: a population-based study”. In: *The Lancet Regional Health–Europe* 24 (2023).

[6] Grégoire Michielin et al. “Clinical sensitivity and specificity of a high-throughput microfluidic nano-immunoassay combined with capillary blood microsampling for the identification of anti-SARS-CoV-2 Spike IgG serostatus”. In: *Plos one* 18.3 (2023), e0283149.

[7] Javier Perez-Saez et al. “Persistence of anti-SARS-CoV-2 antibodies: immunoassay heterogeneity and implications for serosurveillance”. In: *Clinical Microbiology and Infection* 27.11 (2021), 1695–e7.

[8] Andrew S Azman et al. “Vibrio cholerae O1 transmission in Bangladesh: insights from a nationally representative serosurvey”. In: *The Lancet Microbe* 1.8 (2020), e336–e343.

[9] Elsa Lorthe et al. “Epidemiological, virological and serological investigation of a SARS-CoV-2 outbreak (Alpha variant) in a primary school: A prospective longitudinal study”. In: *Plos one* 17.8 (2022), e0272663.

[10] Elsa Lorthe et al. “A SARS-CoV-2 omicron (B. 1.1. 529) variant outbreak in a primary school in Geneva, Switzerland”. In: *The Lancet Infectious Diseases* 22.6 (2022), pp. 767–768.

[11] Bob Carpenter et al. “Stan: A probabilistic programming language”. In: *Journal of statistical software* 76 (2017).

[12] Andrew Gelman and Donald B Rubin. “Inference from iterative simulation using multiple sequences”. In: *Statistical science* 7.4 (1992), pp. 457–472.

[13] William S Hart et al. “Generation time of the alpha and delta SARS-CoV-2 variants: an epidemiological analysis”. In: *The Lancet Infectious Diseases* 22.5 (2022), pp. 603–610.

[14] Simon Cauchemez et al. “Role of social networks in shaping disease transmission during a community outbreak of 2009 H1N1 pandemic influenza”. In: *Proceedings of the National Academy of Sciences* 108.7 (2011), pp. 2825–2830.

[15] Terry C Jones et al. “Estimating infectiousness throughout SARS-CoV-2 infection course”. In: *Science* 373.6551 (2021), eabi5273.

[16] Erik Volz et al. “Assessing transmissibility of SARS-CoV-2 lineage B. 1.1. 7 in England”. In: *Nature* 593.7858 (2021), pp. 266–269.

[17] Carles Bretó et al. “Time series analysis via mechanistic models”. In: *The Annals of Applied Statistics* (2009), pp. 319–348.

[18] Aaron A King et al. “Inapparent infections and cholera dynamics”. In: *Nature* 454.7206 (2008), pp. 877–880.

[19] Joseph C. Lemaitre et al. “Assessing the Impact of Non-Pharmaceutical Interventions on SARS-CoV-2 Transmission in Switzerland”. In: *Swiss Medical Weekly* 21 (May 2020). DOI: 10.4414/smw.2020.20295. (Visited on 09/14/2021).

[20] Caroline Stein et al. “Past SARS-CoV-2 infection protection against re-infection: a systematic review and meta-analysis”. In: *The Lancet* 401.10379 (2023), pp. 833–842.

[21] Thibaut Jombart et al. “Bayesian reconstruction of disease outbreaks by combining epidemiologic and genomic data”. In: *PLoS computational biology* 10.1 (2014), e1003457.

[22] Zoe Swank et al. “A high-throughput microfluidic nanoimmunoassay for detecting anti–SARS-CoV-2 antibodies in serum or ultralow-volume blood samples”. In: *Proceedings of the National Academy of Sciences* 118.18 (2021), e2025289118.

[23] Edward L Ionides, Ning Ning, and Jesse Wheeler. “An iterated block particle filter for inference on coupled dynamic systems with shared and unit-specific parameters”. In: *arXiv preprint arXiv:2206.03837* (2022).
[24] Ning Ning and Edward L Ionides. “Iterated block particle filter for high-dimensional parameter learning: Beating the curse of dimensionality”. In: *J. Mach. Learn. Res.* 24 (2023), pp. 1–76.
[25] Edward L Ionides et al. “Inference for dynamic and latent variable models via iterated, perturbed Bayes maps”. In: *Proceedings of the National Academy of Sciences* 112.3 (2015), pp. 719–724.
[26] Kidus Asfaw et al. “Partially observed Markov processes with spatial structure via the R package spatPomp”. In: *arXiv preprint arXiv:2101.01157* (2021).
[27] Edward L Ionides et al. “Monte Carlo profile confidence intervals for dynamic systems”. In: *Journal of The Royal Society Interface* 14.132 (2017), p. 20170126.
[28] Justin Lessler et al. “Household COVID-19 risk and in-person schooling”. In: *Science* 372.6546 (2021), pp. 1092–1097.
[29] Mélodie Monod et al. “Age groups that sustain resurging COVID-19 epidemics in the United States”. In: *Science* 371.6536 (2021), eabe8372. DOI: 10.1126/science.abe8372. eprint: https://www.science.org/doi/pdf/10.1126/science.abe8372. URL: https://www.science.org/doi/abs/10.1126/science.abe8372.
[30] Jan M Brauner et al. “Inferring the effectiveness of government interventions against COVID-19”. In: *Science* 371.6531 (2021), eabd9338.
[31] Sebastian Walsh et al. “Do school closures and school reopenings affect community transmission of COVID-19? A systematic review of observational studies”. In: *BMJ open* 11.8 (2021), e053371.
[32] Thomas Hale et al. “A global panel database of pandemic policies (Oxford COVID-19 Government Response Tracker)”. In: *Nature human behaviour* 5.4 (2021), pp. 529–538.
[33] Joe Hasell et al. “A cross-country database of COVID-19 testing”. In: *Scientific data* 7.1 (2020), p. 345.
[34] Edouard Mathieu et al. “Coronavirus (COVID-19) Cases”. In: *Our World in Data* (2020). https://ourworldindata.org/covid-cases.
[35] Google LLC. *Google COVID-19 Community Mobility Reports.* (Visited on 04/28/2025).
[36] Simon Galmiche et al. “SARS-CoV-2 Incubation Period across Variants of Concern, Individual Factors, and Circumstances of Infection in France: A Case Series Analysis from the ComCor Study”. In: *The Lancet Microbe* 0.0 (Apr. 2023). ISSN: 2666-5247. DOI: 10.1016/S2666-5247(23)00005-8. (Visited on 05/16/2023).
